# A Revision of North American *Lactura* (Lepidoptera, Zygaenoidea, Lacturidae)

**DOI:** 10.3897/zookeys.846.31953

**Published:** 2019-05-16

**Authors:** Tanner A. Matson, David L. Wagner, Scott E. Miller

**Affiliations:** 1 Department of Ecology and Evolutionary Biology, University of Connecticut, Storrs, Connecticut 06269-3043, USA University of Connecticut Storrs United States of America; 2 Department of Entomology, National Museum of Natural History, Smithsonian Institution, Washington DC 20013-7012, USA National Museum of Natural History, Smithsonian Institution Washington United States of America

**Keywords:** CO1, DNA barcodes, gum bully, subspiracular gland, Sapotaceae, Sideroxylon, tropical burnet moths

## Abstract

The *Lactura* Walker, 1854 fauna north of Mexico is revised. Six species are documented, one new species *Lacturanalli* Matson & Wagner, **sp. n.** is described, and two new synonymies are proposed: *Lacturapsammitis* (Zeller, 1872), **syn. n.** and *L.rhodocentra* (Meyrick, 1913), **syn. n.** One new subspecies *Lacturasubfervenssapeloensis* Matson & Wagner, **ssp. n.** is also described. Adult and larval stages, male and female genitalia, are illustrated, a preliminary phylogeny is presented based on nuclear and mitochondrial data, distribution records provided for verified specimens, and the biology and life history for each species is briefly characterized. Phylogenetic analyses, larval phenotypes, and life history information reveal that much of the historic taxonomic confusion rampant across this group in North America traces to the phenotypic variation in just one species, *L.subfervens* (Walker, 1854).

## Introduction

Lacturidae (tropical burnet moths) are a tropical and sub-tropical family of Zygaenoidea. Prior to the family description by Heppner (1995), members of this group had been classified in Plutellidae, Zygaenidae, and Yponomeutidae (Epstein et al. 1998). Although the current global diversity is around 140 species, Heppner (2008) estimates the world fauna to be in excess of 250 species. This disparity traces to large numbers of undescribed tropical species. In North America, taxonomic understanding of the family has been hamstrung by confounding phenotypic overlap in color and pattern among many species (Heppner 2008, Matson and Wagner 2017).

The most recent checklist of North American Lepidoptera (found north of Mexico) recognizes six species of *Lactura* (Heppner and Duckworth 1983): *Lacturapupula* (Hübner, [1831]), *Lacturasubfervens* (Walker, 1854), *Lacturapsammitis* (Zeller, 1872), *Lacturabasistriga* (Barnes and McDunnough, 1913), *Lacturaatrolinea* (Barnes and McDunnough, 1913), and *L.rhodocentra* (Meyrick, 1913). To this set, we add *Lacturarubritegula* (Matson & Wagner, 2017) from the southern Hill Country of central Texas; *L.nalli* sp. n. from the Rio Grande Valley of southern Texas; and *Lacturasubfervenssapeloensis* ssp. n. from Florida and coastal Georgia. The taxonomic identity and validity of Nearctic *Lactura* names – long conflated in literature, institutional collections, and digital databases – are resolved. Much of the confused species-level taxonomy of the genus traces to *L.subfervens*, a widespread and phenotypically variable entity whose forewings range from mottled smoky red to almost entirely white, with variously developed rows of antemedial and postmedial spots (Matson and Wagner 2017).

Our collections of wild *Lactura* caterpillars and reared ex-ova cohorts from across the southeastern US and Texas have revealed six distinct larval phenotypes. These were found to align with CO1 haplotype clusters (Matson and Wagner 2017) and are now additionally supported by nuclear data presented here. Using larvae, life history information, and molecular data, we redescribe five species, describe one new species and one new subspecies, and synonymize *L.psammitis* and *L.rhodocentra*. We provide redescriptions and diagnoses for adults, brief descriptions of living larvae, and life history notes for each species; taxonomic keys and images for both larvae and adults, keys for male and female genitalia; and a phylogeny based on seven nuclear loci and two mitochondrial CO1 loci.

## Materials and methods

### Morphology

Fresh adult collections were obtained by light trapping with UV and mercury-vapor lights. Larvae were collected from *Sideroxylon* (Sapotaceae). Additional larval specimens were reared from ova deposited by gravid females. We examined the Nearctic *Lactura* holdings of the following museums:

**CC** College of Charleston, Charleston, SC

**ECK** Edward C Knudson, personal collection, Houston, TX

**FEM** Frost Entomological Museum, Penn State University, State College, PA

**FMNH** McGuire Center for Lepidoptera and Biodiversity, Florida Museum of Natural History, Gainesville, FL

**JKA** James K Adams, personal collection, Dalton, GA

**JRM** James R McDermott, personal collection, College Station, TX

**KSU-MEPAR** Kansas State University–Museum of Entomological and Prairie Arthropod Research, Manhattan, KS

**MEM** Mississippi Entomological Museum, Mississippi State, MS

**NHMUK** The Natural History Museum London, London, UK

**TAMUIC** Texas A&M University Insect Collection, College Station, TX

**UCMS** Biodiversity Research Collections, University of Connecticut, Storrs, CT

**USNM**National Museum of Natural History, Washington DC (including primary types)

Photographs from BugGuide, iNaturalist, Moth Photographers Group, and Barcode of Life Database (Ratnasingham and Hebert 2007), through June of 2018, were examined and occurrence data added to an Excel file (Suppl. material 1). Images of types in the NHMUK were provided by Maia Vaswani. Adult redescriptions are based on 986 specimens: *L.pupula* (n = 231), *L.atrolinea* (n = 138), *L.basistriga* (n = 97), *L.subfervens* (n = 474), *L.subfervenssapeloensis* (n = 21), and *L.rubritegula* (n = 25). Several paratypes of *L.subfervenssapeloensis* were added late and are not reflected in the phenology and distribution figures. Forty-two genitalic slides of *Lactura* were examined: 29 from the USNM and 13 prepared by Tony Thomas for this study. SimpleMappr (http://www.simplemappr.net) was used to generate the geographic distribution point map (Shorthouse 2010). Types for *L.nalli* are deposited at TAMUIC, UCMS, and USNM. Types for *L.subfervenssapeloensis* are deposited at CC, FMNH, UCMS, USNM, and JKA.

### Barcoding

CO1 barcodes for North American *Lactura* were compiled from holdings in the following institutions and personal collections: (CNC) Canadian National Collection of Insects, Arachnids, and Nematodes, FMNH, JRM, MEM, TAMUIC, UCMS, and USNM. We had access to 116 North American *Lactura* COI barcode submissions: for each of these, we examined the associated voucher specimen or image. 93 of the 116 CO1 barcodes were used (failed sequences excluded) to generate a neighbor-joining tree, using the default Kimura-2P model in the Barcode of Life Project (BOLD) (http://www.boldsystems.org) (Ratnasingham and Hebert 2007). DNA extraction, PCR amplification, and CO1 barcode sequencing were performed at the Canadian Centre for DNA Barcoding (Centre for Biodiversity Genomics – University of Guelph) using their standard Sanger sequencing protocols (Wilson 2012). Data for 93 sequences representing ten barcode clusters (putative species) have been released on GenBank (accession numbers MK505610-MK505668), and more data, including images, are available on BOLD, (accessible in the dataset LACTURA1 using a DOI (dx.doi.org/10.5883/DS-LACTURA1)).

### Multi-gene analysis

Seven single-copy genes capable of resolving phylogenetic relationships of Lepidoptera were sampled (5508 bp total): cytochrome c oxidase subunit 1 (CO1) from the mitochondrial genome and elongation factor–1 α (EF–1α), glyceraldehyde–3–phosphate dehydrogenase (GAPDH), isocitrate dehydrogenase (IDH), cytosolic malate dehydrogenase (MDH), sorting nexin–9–like protein (Nex9), and ribosomal protein S5 (RpS5) from the nuclear genome (Cho et al. 1995, Fang et al. 1997, Mitchell et al. 2006, Wahlberg and Wheat 2008, Zahiri et al. 2011, Rota et al. 2016, Regier et al. 2017). Both CO1 and EF–1α were sequenced in two parts making for a total of nine loci. All DNA extractions were performed using the protocol and material from Macherey Nagel’s NucleoSpin Tissue 250 kit. A single leg was taken from each specimen. Once extracted, DNA was stored in a ~4 °C refrigerator until needed for PCR. The PCR profiles and primers outlined in Wahlberg and Wheat (2008) were used. PCR products were sent to Macrogen USA Inc. (Rockville, Maryland) for sequencing. Sequence chromatograms were visually inspected for base call errors and heterozygous loci in Geneious v. 8.1.9 (Kearse et al. 2012). Sequences were then exported to FASTA files and visually aligned to reference lepidopteran sequences for each locus using AliView v. 1.18 (Larsson 2014). GenBank accession numbers and voucher codes for all sequences used in the molecular analysis are given in Table 1.

**Table 1.** Specimen voucher data and GenBank accession numbers for samples used in phylogenetic analysis. Dash indicates DNA markers that did not amplify.

Voucher Code	Genus Species	CO1 Begin	CO1 End	EF1-a Begin	EF1-a End	RpS5	MDH	Nex9	IDH	GAPDH
TAM0012	*Lacturarubritegula*	MH536189	MH536189	MH553373	MH553373	MH545982	MH545969	–	MH545979	MH545966
TAM0006	*Lacturarubritegula*	MH536196	MH536196	MH553372	MH553372	MH545989	MH545978	–	–	–
TAM0013	*Lacturabasistriga*	MH543321	–	MH553366	–	MH545986	MH545973	–	–	–
TAM0005	*Lacturabasistriga*	–	–	MH553365	–	MH545985	MH545972	–	–	–
TAM0011	*Lacturanalli*	MH536192	MH536192	MH553371	MH553371	MH545987	MH545974	–	MH545980	MH545967
TAM0007	*Lacturasubfervens*	MH536190	MH536190	MH553370	MH553370	MH545983	MH545970	MH545991	–	–
TAM0003	*Lacturasubfervenssapeloensis*	MH536193	MH536193	MH553367	–	–	MH545975	MH545993	–	–
TAM0010	*Lacturasubfervenssapeloensis*	MH536194	MH536194	MH553368	–	MH545988	MH545976	–	–	–
TAM0004	*Lacturaatrolinea*	MH536188	MH536188	MH553363	–	MH545981	MH545968	MH545990	–	–
TAM0008	*Lacturaatrolinea*	MH536191	MH536191	MH553364	–	MH545984	MH545971	MH545992	–	–
TAM0009	*Lacturapupula*	MH536195	MH536195	MH553369	–	–	MH545977	MH545994	–	–
MM00180	*Anticrates* sp.	GU828624	GU828422	GU828959	–	GU830631	GU830332	–	–	–
SEM-06-5644	*Strigiveniferavenata*	GU828864	–	GU829167	GU829429	GU830811	GU830556	–	GU830242	–
MM00312	*Adscitastatices*	GU828630	GU828428	GU828965	GU829251	GU830634	GU830338	–	GU830017	GU829769
06-srnp-33730	*Dalceridesgugelmanni*	GU828533	GU828335	GU828879	–	–	GU830257	–	GU829924	–

### Phylogenetic analysis

Outgroup Zygaenoidea included *Adscitastatices* Linnaeus (Zygaenidae), *Strigiveniferavenata* Aurivillius (Limacodidae), and *Dalceridesgugelmanni* Dyar (Dalceridae), as well as another lacturid, *Anticrates* Meyrick. Sequences were partitioned by locus and codon position. The best-fit model of nucleotide substitution for each partition was found using the Akaike Information Criterion in PARTITIONFINDER2 (Lanfear et al. 2016) on the CIPRES web server (Miller et al. 2010). IQTREE v. 1.6.6 (Nguyen et al. 2014) was used to infer the likelihood trees and calculate bootstrap values (1,000 replicates). MRBAYES v. 3.2.6 (Huelsenbeck and Ronquist 2001, Ronquist and Huelsenbeck 2003) was used for the Bayesian analyses. Bayesian analyses were run for 10 million generations, with every 1000^th^ generation sampled. Clade robustness was estimated by posterior probabilities in MrBayes. Stationarity of MCMC parameters were assessed with TRACER v 1.6.0 (Rambaut et al. 2014).

## Results

### Generic diagnosis and description

#### Lactura

Taxon classificationAnimaliaLepidopteraLacturidae

Walker, 1854: 485

Figs 1–2 , 3 , 4 , 5, 6 , 7, 8 , 9–14 , 50–51 , 52–53 , 63 , Table 1

##### Type-species.

*Lacturadives* Walker, 1854: 485.

Heppner (1997) successfully applied to conserve the widely used name *Lactura* Walker, 1854 and suppress the generic names *Eustixis* Hübner, [1827–31] and *Mieza* Walker, 1854 (ICZN 1999). Heppner’s justification is discussed below, in the treatment for *Lacturapupula*. Generic synonyms can be found in Nye and Fletcher (1991) and Heppner (1995).

**Figures 1, 2.**
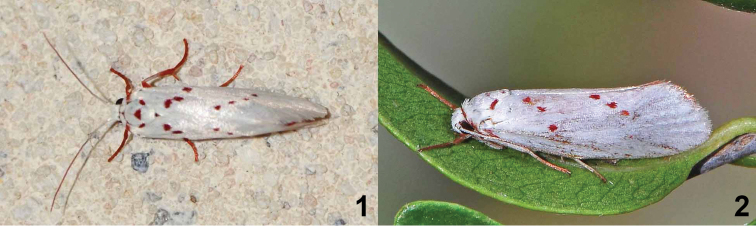
*Lactura* resting posture. **1***Lacturarubritegula* dorsal view **2***Lacturanalli* lateral view.

**Figure 3.**
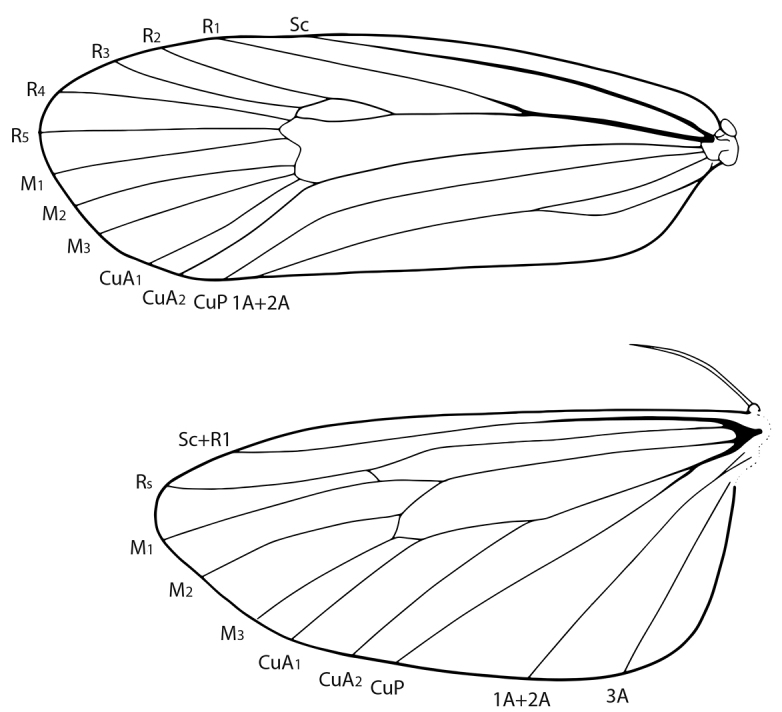
*Lactura* venation (based on venation of *L.pupula*).

**Figure 4.**
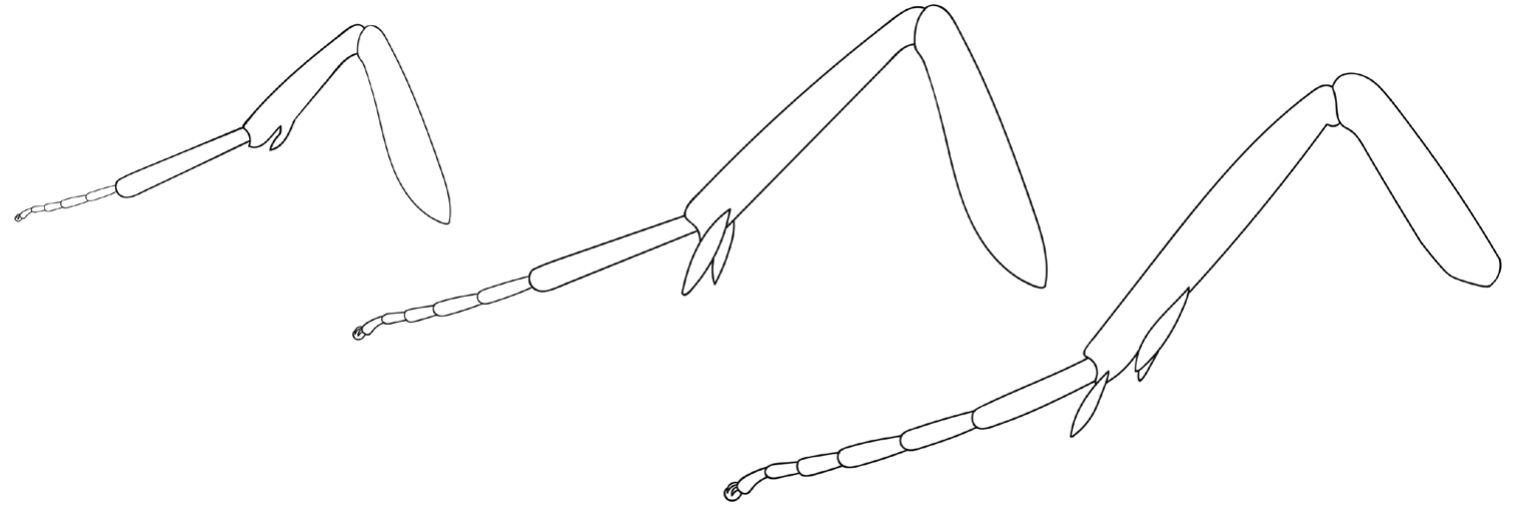
Adult *Lactura* legs (based on *L.pupula*).

**Figures 5, 6.**
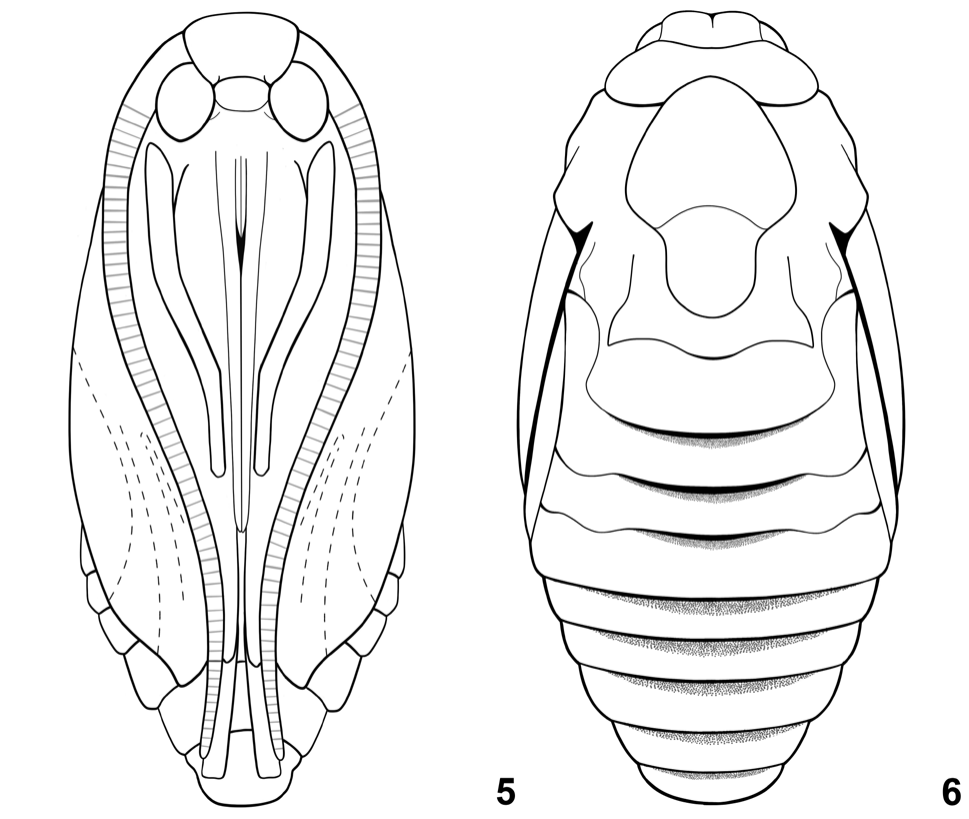
*Lactura* pupa (based on *L.nalli*). **5** Ventral view **6** dorsal view.

**Figures 7, 8.**
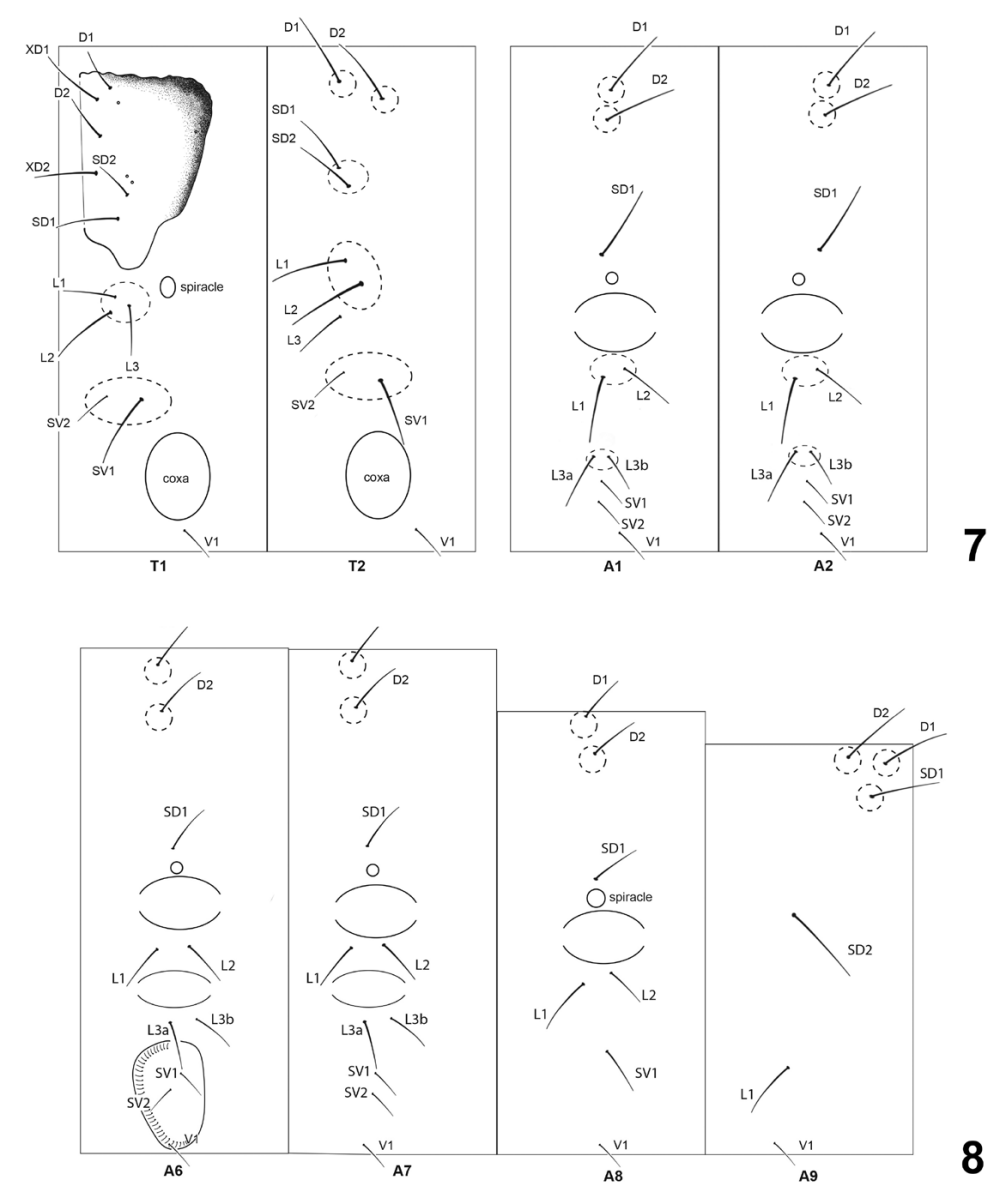
*Lactura* chaetotaxy (based on *L.pupula*). **7** Segments T1–T2, A1–A2 **8** segments A6–A9.

**Figures 9–14.**
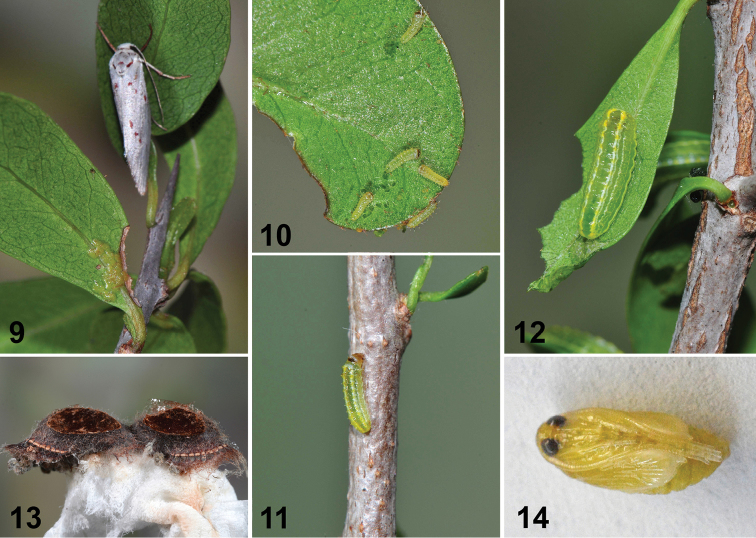
Life history of *Lacturanalli*. **9** Egg cluster (near petiole of leaf below resting female) **10** first instar **11** second instar **12** penultimate instar **13** cocoons **14** pupa.

##### Generic description for adult Nearctic *Lactura*.

Medium-sized (wingspan 17–26 mm) with respect to other members of the family. Body salmon red. **Head.** Antenna filiform in both sexes; labial palpus porrect, maxillary palpus small. **Thorax.** Adult forewing elongate and subquadrangular, widest at 2/3, and typically satin white or white with speckled brown or red maculation. Oblique antemedial and postmedial rows of red or black spots often present. Apex broadly rounded and little differentiated from outer margin, bounded within R4 and R5; likewise, tornus poorly differentiated. Forewing discal cell with areole; R, M, and Cu veins sessile; CuA veins running to tornus; A1 and A2 fused from basal quarter to inner margin; A3 absent. Hindwing oval quadrate and uniformly pink-red or red-orange with elongate fringe scales. Frenulum 1/5 the size of hindwing length; wing apex poorly defined, bounded within Rs and M1; R, M, and Cu veins sessile; vannal area greatly enlarged; distinct fold between A1+A2 and A3. Venation as in Fig. 3. Tibial spur formula 0–2–4. **Abdomen.** Orange-red, midventer paler, often rusty white. One to two sets of androconial hairpencils within intersegmental folds between A7 and A8 (*L.pupula*, some *L.subfervens*), or A6 and A7 and A7 and A8 (*L.atrolinea*, *L.basistriga*, *L.nalli*, *L.rubritegula*, some *L.subfervens*). Cluster of elliptic scales anterior to spiracle and dorsad of hairpencil. At rest, the hairpencil overlays this scale cluster (presumably loading pheromone onto the hairpencil). **Male genitalia** (Figs 23–30). Uncus narrow, strongly down-curved and cylindrical, ending in apical spine. Tegumen pulmonate with strong medial crease. Valva elongate-oval (2.0–2.5 × longer than wide); costa covered in long setae, concave at distal third with broadly rounded apex; outer margin with shorter, thicker scales; lateral lobe of juxta with spiniform setae (more sclerotized and robust with thicker setae in *L.subfervens*, *L.basistriga*, and *L.rubritegula*). Vinculum narrow, U-shaped, subquadrangular. Aedeagus exceeding length of valva (~4.5 × longer than wide) with broadly rounded base and gaping concave aperture at apex. Aperture oblique in *L.subfervens*, *L.basistriga*, *L.nalli*, and *L.rubritegula*. Apical thumb-like process present in *L.pupula* and *L.atrolinea*. **Female genitalia** (Figs 37–43). Papillae anales ca. 4 × times longer than broad with dorsal sclerotized rim conjoined with posterior apophyses. Apophyses rod-like; posterior apophysis ca. three times longer than anterior apophysis. Antrum modestly differentiated, weakly sclerotized, hat-shaped to quadrangular. Ductus bursae distally linear and then variously drawn into series of coils (the number of which is species specific), without accessory diverticula; diameter gradually increasing to corpus bursae and coils becoming more closely drawn together anteriorly. Ductus seminalis attached proximate to antrum. Corpus bursae longer than broad with signa arranged in four hemispherical lobes with dentate interior projections. Signa positioned in two transverse groups; posterior signa half again the size of anterior signa. Accessory pouch sometimes present at anterior end of corpus bursae (*L.atrolinea*, *L.basistriga*, *L.nalli*).

##### Color and habitus of living final instar larva

(Figs 44–49). Caterpillar shiny, usually brightly colored, somewhat tacky to the touch, and resembling small limacodids but with short, crochet-bearing prolegs and greater degree of translucence. Ground color green, with brown, black, or green dorsum. Dorsal and subdorsal setae borne from yellow, orange, black, and in some cases blue (*L.atrolinea*) translucent warts. Prothoracic plate well differentiated, divided medially; head partially retracted into thorax. Head dark brown or black, strongly sclerotized with long antennae.

##### Final instar larva

(Figs 7, 8). Description based on *L.pupula*. **Head.** Somewhat prognathous; anterior half more strongly sclerotized; darkly melanized patches to either side and through frons; larger patch, that includes stemmata, extending to level of P1 seta; labrum (0.2 mm long x 0.4 mm wide) with margins rounded and a medial notch; clypeus bulging; antenna longer than labial palpus; head setae generally short except for anteriormost seta of AF, A, S, and SS groups. **Thorax** (Fig. 7). **T1**: Prothoracic shield well developed and strongly sclerotized along mesal and posterior edges; XD1, XD2, SD1, and D2 longest setae on prothorax; D1 and D2 shifted near anterior edge of pronotum, D2 nearly twice as long as D1 and arising midway between and just posteriad of XD1 and XD2; MXD1 included on prothoracic shield directly posterior to D2. Three pits on prothoracic shield; uppermost posteriad of XD1, and two closely set pits dorsad of SD2. Prothoracic spiracle ovate, diameter 0.3 × larger than anterior abdominal spiracles. L group trisetose on raised fleshy wart ventroanteriad to spiracle; two SV setae aligned horizontally, each borne from fleshy wart. **T2**: All setae borne from raised fleshy warts that become increasingly sclerotized to point where seta issues, with size of wart and degree of sclerotization decreasing ventrad. D1, D2, and SD2 nearly in line; D1 and D2 subequal in length and sharing common ridge; SD1 and SD2 also sharing common ridge. Two dorsal L setae sharing a common fleshy wart and lower L seta positioned midway between upper L setae and SV setae. SV1 and SV2 on shared lateral swelling. **T3**: As in T2 except with only two L setae; uppermost L seta shifted upward and sharing protuberance with SD setae. **Abdomen** (Figs 7, 8). First eight segments with elliptical subspiracular gland and smaller subventral swelling (that may also be glandular). Setae mostly aligned along spiracular meridian and borne from clear, raised, wart-like fleshy swellings. **A1**: D1 and D2 on common swelling, transversely aligned with SD1 and spiracle; SD1 above spiracle; SD2 not observed (presumably microscopic); spiracle circular. L1 and L2 on same swelling below subspiracular gland; L2 (evidently) two-thirds length of L1 and located dorsoposteriad; L3 paired on separate swelling; SV1, SV2, and V1 in line, SV2 less than half size of SV1; V1 seta directed mesad. **A2**: Same as A1. **A3–A6**: D1, D2, SD1, L1, and L2 as on A1 and A2. L3 setae horizontally positioned beneath subventral swelling. Proleg with SV and V setae; 32–35 crochets in biordinal, mesal semi-circle along inner and posterior margin. **A7**: As in previous segments. SD1 dorsoposteriad to spiracle. V1 reduced or concealed in fold of subventral swelling. **A8**: D2 posteriolaterad to D1; both lightly sclerotized; spiracle nearly circular, 0.12 mm in diameter; 3 L setae. **A9**: D2 anterior to D1; D1, D2, and SD2 on enlarged wart; D1 pinaculum sclerotized; single L seta. **A10**: modestly sclerotized; four pairs of setae along caudal margin; 28–30 crochets; anal plate with melanized patch to either side of midline.

##### Pupa

(Figs 5, 6, 14). Subobtect, broadest through abdominal segments and modestly dorsoventrally flattened; antennae, legs, and wings loosely fused to body; setae very short and inconspicuous. Labial palpi visible; proboscis ending beyond protibia; caudal margin of metathorax curved upward and forming low ridge over dorsum of first abdominal tergite. Forewing apex distinctly falcate; hindwing exposed with vannal lobe extended upward toward spiracles. Pro-, meso-, and metatarsus fully visible; mesotibia ending between anterior of A5 to caudal margin of A7; metatibia and antenna subequal, and of variable length, extending to caudal margin of A7 to exceeding A10. Abdomen swollen through spiracular and subspiracular areas (perhaps as a consequence of larval subspiracular glands and subventral swellings); A2–A8 with supraspiracular recess and narrow band of rearward-directed teeth along anterior margin extending from midline nearly to supraspiracular area (presumably assisting in eclosion). A10 smooth with slight cleft and posterolateral protrusions; cocoon cutter, cremaster, and pseudocremaster absent.

##### Cocoon

(Fig. 13). Parchment-like, upper 4/5 of cocoon thick, tough, and carapace-like. Outer edge of cocoon with darkly stained, irregular, reticulate skirt which serves to anchor cocoon to substrate. Floor of cocoon tan, parchment-like; U-shaped operculum at one end tears free at eclosion. Pupa not extruded at eclosion.

##### Distribution and biology

(Figs 9–14, 50–53). North American *Lactura* are found in woodlands, scrublands, and thicket communities from Kansas, Missouri, Illinois, and Kentucky, south through Texas and the Gulf States, east to coastal South Carolina, Georgia, and the whole of Florida, continuing into the Caribbean and Mexico (Figs 52, 53). All species treated here feed exclusively on *Sideroxylon* spp. (Sapotaceae) and overwinter as prepupae inside a tough reddish to brownish cocoon spun in leaf litter (Fig. 13). Across much of their range in the United States, *Lactura* are primarily univoltine, tied to new growth as larvae. Species of the Rio Grande Valley and southern Florida occur nearly year-round, at least in small numbers, if new leaves are present. In captivity larvae typically feed from leaf undersides, with the head partially retracted into the prothorax. The fecal pellets are characteristically spherical, lacking any hint of the concave depression common to the pellets of limacodids and megalopygids.

*Lactura* are difficult to rear, often molding or desiccating as prepupae. We have had modest success rearing larvae in vials with a deep (3–5 cm), occasionally moistened, layer of peat or coir.

Larvae secrete a tacky exudate from their integument to which feculae adhere and often remain attached until the next molt. We suspect the exudate is a deterrent to ants and other invertebrates, as such sticky secretions have been shown to be in the caterpillars of the related Dalceridae (Epstein et al. 1994). When *Lactura* caterpillars are threatened, they evert transparent, balloon-like vesicles from the side of their body (Figs 50, 51) that secrete a sticky, mucilaginous fluid. As far as known, these structures are present in all Nearctic species, but especially apparent in *L.pupula*. It is unclear how prevalent these subspiracular glands and associated behaviors are among other lacturids because the life histories for most species remain unknown.

We suspect adult *Lactura* sometimes disperse from their natal colonies, given that several collection records appear to be out of range, e.g., a single *L.rubritegula* from Houston, Texas (Fig. 52) (Matson and Wagner 2017). Individuals have also been taken or photographed outside the range of their respective *Sideroxylon* hosts. Adults have a well-developed proboscis, but we have no records of adults feeding at flowers. Despite their bright, seemingly aposematic coloration, the adults of Nearctic species are thought to be exclusively nocturnal.

### Key to *Lactura* adults found north of Mexico

**Table:** 

1	White forewing with black dashes, spots, or streaks; light red scales on vertex	**2**
–	White forewing with series of red or brown dashes, spots, or streaks; white scales one vertex	**3**
2	Forewing with black scaling over veins in upper half of forewing extending around the apex and terminating about tornus; two oblong antemedial spots and three oblong postmedial spots; absent from southern Texas	***Lacturapupula* (Fig. 22)**
–	Forewing without black scaling over veins; contiguous series of black antemedial and postmedial spots and black subcostal dash; from southern Texas	***Lacturaatrolinea* (Fig. 18)**
3.	Tegula with inconspicuous red scale patch at base (below plane of forewing), otherwise white; forewing entirely white (apart from antemedial and postmedial spots); red subcostal dash often present at base of FW; from southern Texas	***Lacturabasistriga* (Fig. 20), *L.nalli* (Figs 2, 21)**
–	Tegula basally and medially red (extending well above plane of forewing); forewing with or without scattered red or brown scales (apart from antemedial and postmedial spots); subcostal dash absent at base of FW; absent from extreme southern Texas	**4**
4	Forewing with scattered red/brown scales (variable in density); antemedial and postmedial spots present but sometimes reduced; patagium white; widespread across portions of Midwest and southeastern US	***Lacturasubfervens* (Figs 15–17)**
–	Forewing without scattered red/brown scales; antemedial and postmedial spots present; patagium with red scales near collar; west central Texas	***Lacturarubritegula* (Figs 1, 19)**

### Key to late instars of *Lactura* found north of Mexico

**Table:** 

1	Dorsum darkly pigmented: gray, black, or brown	**2**
–	Dorsum without gray, black, or brown pigmentation	**3**
2	From extreme southern Texas (Hidalgo and Cameron Counties)	**4**
–	Absent from extreme southern Texas	**5**
3	Ground color green, green semitransparent dorsum; from western end of Rio Grande Valley of Texas	***Lacturanalli* (Figs 48, 51)**
–	Ground color frosty, white stripes over dorsum; widespread across southern Midwest and southeast US, absent from Rio Grande Valley of Texas	***Lacturasubfervens* (Fig. 45)**
4	Dorsum with yellow middorsal stripe and green addorsal stripe; conspicuous metallic blue warts	***Lacturaatrolinea* (Fig. 47)**
–	Variable dark coloration over dorsum interrupted by wavy, white addorsal stripe; conspicuous yellow warts	***Lacturabasistriga* (Fig. 49)**
5	Prominent, white to orange addorsal stripes with embedded orange warts; widespread across Midwest and southeastern US	***Lacturapupula* (Figs 44, 50)**
–	No orange stripping or warts; broad, cinnamon-brown middorsum outwardly edged by black addorsal stripes; from west central Texas	***Lacturarubritegula* (Fig. 46)**

### Key to male genitalia of *Lactura* found north of Mexico

**Table:** 

1	Lateral lobe of juxta lightly sclerotized with 20–30+ spiniform setae; aedeagus with large, broadly concave aperture and thumb-like distal process	**2**
–	Lateral lobe of juxta heavily sclerotized with 10–20+ thickened spiniform setae; aedeagus with obliquely concave aperture, thumb-like distal process absent	**3**
2	Uncus with strong medial constriction in basal third; broadly cylindrical to apex	***L.atrolinea* (Fig. 25)**
–	Uncus robust and tapering with slight medial constriction in basal third; narrowly cylindrical to apex	***L.pupula* (Fig. 26)**
3	Uncus basally quadrangular	***L.subfervens* (Fig. 24, 27)**
–	Uncus basally cordiform	***L.basistriga* (Fig. 28), *L.rubritegula* (Figs 29, 30), *L.nalli* (Fig. 23)**

### Key to female genitalia of *Lactura* found north of Mexico

**Table:** 

1	Anterior accessory pouch present on corpus bursae; 4–6 coils in ductus bursae; signa ca. half diameter of corpus bursa	**2**
–	Anterior accessory pouch absent on corpus bursae; 6–12 coils in ductus bursae; signa ca. quarter diameter of corpus bursae	**3**
2	Diameter of ductus bursae coils subequal throughout; anterior accessory pouch connected to corpus bursae by broad opening	***L.atrolinea* (Fig. 39)**
–	Diameter of ductus bursae coils increasing in size anteriorly, anteriormost coil ca. 3× diameter that of posteriormost; anterior accessory pouch connected to corpus bursae by narrow opening	***L.basistriga* (Fig. 37), *L.nalli* (Fig. 38)**
3	Six to eight coils in ductus bursa	***L.rubritegula* (Fig. 41)**
–	Nine to twelve coils in ductus bursa	***L.subfervens* (Fig. 42–43), *L.pupula* (Fig. 40)**


#### Lactura pupula

Taxon classificationAnimaliaLepidopteraLacturidae

(Hübner)

Figs 22 , 26 , 31 , 40 , 44 , 50 , 52 , 54–56 , 63 , Table 1

Eustixis pupula Hübner, [1831]: 24. Type locality: Georgia, USA. Type material: presumably lost
Eustixis leata Geyer, 1832: 5. Type locality: Unknown. Type material: not examined
Mieza igninix Walker 1854: 527. Type locality: St. John’s Bluff, E. Florida, USA. Type material: not examined
Enaemia crassivenella Zeller 1872: 563. Type locality: Texas, USA. Type material: not examined for this study, but seen earlier by Miller (Miller and Hodges 1990)
Enaemia crassinervella Slosson, 1896: 86; misspelling

##### Notes.

*Lacturapupula* was first described as *Eustixispupula* in vol. 3 of Hübner’s Zuträge zur Sammlung exotischer Schmetterlinge (1827–1831). The original description was published subsequent to Hübner’s death, and the approximate date of description has been inferred to be 1831 (Heppner 1997). The original description is vague, weakly informative, and the taxonomy confusing. The type is presumably lost; however, the illustration in Hübner’s manuscript is unambiguous, and assignable to *L.pupula* as recognized in this work. Years prior, Hübner (1823) gave the name *Eustixiapupula* to the spotted peppergrass moth, a well-known crambid. Both *Eustixiapupula* and *Eustixispupula* were described from North America, spelled similarly, and both of their descriptions contain the Latin phrase, “*Phalaenavera*, *Lithosiageometriformis*.” It is debated to what extent this is intentional and whether Hübner considered the two congeners. Whatever the case, *Eustixiapupula* and *Eustixispupula* are entirely different moths (representing two different superfamilies) and each name remains available.

A few decades later Walker (1854), established the generic names *Lactura* and *Mieza*. Both *Miezaigninix* and *Eustixislaeta* were placed in the latter genus and considered subjective synonyms of *Eustixispupula* (Walsingham 1914) (Nye and Fletcher 1991). In literature, *Eustixis* is largely ignored, perhaps because of its near homonymy with *Eustixia*; *Mieza* as well became infrequently used. All authors after Walsingham (1914) used *Lactura*, by reason of page priority in Walker (1854) and *Mieza* has since been suppressed (Heppner 1997) (ICZN 1999).

*Enaemiacrassivenella* (Zeller, 1872) and *Enaemiacrassinervella* (Slosson, 1896) are both synonyms of *L.pupula*—Slosson (1896) was undoubtedly referring to “*crassivenella*” and “*crassinervella*” was simply a lapse in spelling.

##### Diagnosis.

Forewing pattern instantly distinguishes this species from its congeners. The most notable difference is the black streaking along the veins of the forewing, and two oblong antemedial spots and three oblong postmedial spots in the lower half of the forewing. The postmedial spots are arranged in a triangular pattern with the lower distal spot touching the inner margin. Female genitalia have 9–10 distal spirals in the ductus bursae. The larva's orange verrucae on white to orange addorsal stripes distinguishes it from all other Nearctic *Lactura*.

##### Description adult

(Fig. 22). Forewing length: 9–13 mm (n = 231). **Head.** Light red to orange over vertex transitioning to white over frons. Labial palpus slightly porrect to straight, brick red at base and black apically, length subequal to eye diameter. Antenna filiform, 0.6 length of forewing; shiny, white above, fuscous below. **Thorax.** Patagium mostly white, with black basal scales forming contrasting collar behind head. Tegula with small ventral black basal patch; white medially and shiny black apically. Large medial mesothoracic and metathoracic black spots. Coxa and femur with red dorsal surface and light red to pale white ventral and lateral surfaces; procoxa with basal mixture of red and black scales; pro- and mesotibia and pro- and mesotarsus black or fuscous dorsally and fuscous red ventrally. Metatibia light red; metatarsus fuscous red. **Forewing.** White with black scales over veins; black scaling extending around apex and terminating about tornus; variable in thickness. Two oblong antemedial spots and three oblong postmedial spots in lower half. Postmedial spots arranged in triangular pattern with lower distal spot elongate and touching inner margin. Costal margin black along basal 1/3 of wing. Underside light red. Fringe scales light red, rarely with admixture of black scales. **Hindwing.** Uniformly light red to dark orange, above and below, with concolorous fringe scales. **Abdomen.** Dorsum and sides brick red; venter rusty white. One pair of subventral intersegmental hairpencils (consisting of 40–60 androconial scales) between A7 and A8 (Fig. 31). **Male Genitalia** (Fig. 26) (n = 7). Uncus strongly down-curved; basally quadrangular and medially constricted through basal third; cylindrical, tapering to apex, ending in apical spine. Valva elongate-oval, ca. 2 × longer than wide, concave along distal third of costal margin; broadly rounded at apex; lateral lobe of juxta with 20–30+ setae, similar to *L.atrolinea*, but setae shorter and bearing less robust spiniform setae than those of other congeners. Vinculum narrow, U-shaped, subquadrangular. Aedeagus cylindrical, exceeding length of valva; base broadly rounded; apex with broad concave aperture and thumb-like process twice as long as wide. **Female genitalia** (Fig. 40) (n = 3). Papillae anales ca. 3.5 × times longer than broad with dorsal sclerotized rim fused with posterior apophyses. Ductus bursae with 9–11 coils, posterior two coils more open and extended than anterior coils; diameter mostly uniform; coil diameters more or less uniform with anteriormost coil slightly larger than others. Quadrate signa reduced in size compared to other species treated here; lobes fused in two of three preps. Corpus bursae without anterior accessory pouch.

##### Description of living final instar

(Figs 44, 50). Ground color pale and mostly unpigmented to mint green, translucent below spiracles. Thin, white to orange middorsal stripe, edged with thick addorsal stripe; prominent white to orange dorsal stripe with orange verrucae that bear D1 and D2 setae; verrucae on A8 more pronounced than others. Thick black dorsolateral stripe divided by white pinstripe on T2–A8. Thick, white supraspiracular stripe best developed on T3–A8. Spiracular stripe thin and white; interrupted by light orange spiracles. The extent of orange coloration is reduced in larvae from Texas, where orange is mostly restricted to the thorax and A7-A9, and the stripe running through the dorsal verrucae is more given to white than orange.

##### Distribution and biology.

*Lacturapupula* occurs in woodlands, thickets, scrublands, back dune and coastal strand communities, and along forest edges of central Texas northward in Midwest to Nebraska and Illinois, and eastward to South Carolina and the whole of Florida (Fig. 52). The moth flies from February to October (southward) and is often abundant during its peak flight in March and April in Florida. Southward it is multivoltine, especially in southern Florida where it flies nearly year-round (Figs 54–56). Our host plant records are from gum bully (*Sideroxylonlanuginosum*) and tough bully (*Sideroxylontenax*); Kimball (1965) also lists saffron plum (*Sideroxyloncelastrinum*). Larvae co-occur on the same hostplants with those of *L.subfervens* over much of its range.

In central and west-central Texas, larvae show a reduction in the amount of dorsal orange maculation. A close relative of *L.pupula*, based on CO1 data in BOLD (BOLD: ACN5528), occurs in Tamaulipas, Mexico. It would be worthwhile to do more sampling in south Texas and northern Tamaulipas to better delineate the ranges of the two moths. *Lacturapupula* is rapidly expanding its range westward in Texas. In 2019, the first records of adults were made in Austin (Travis County), Boerne (Kendall County), and Camp Wood (Edwards County)—all at sites that have been regularly sampled over the past decade. Larvae were found in great numbers in the first two of these counties in April of this year.

#### Lactura subfervens

Taxon classificationAnimaliaLepidopteraLacturidae

Walker

Figs 15–17 , 24 , 27 , 33 , 36 , 42–43 , 45 , 53 , 58 , 59 , 63 , Table 1

Mieza subfervens Walker 1854: 527. Type locality: Texas, USA. Type material: NHMUK, TYPE – BMNH(E) 819792
Lactura psammitis , Zeller 1872: 562; syn. n. Type locality: Texas, USA. Type material: NHMUK, TYPE – BMNH(E) 1377410
Lactura rhodocentra , Meyrick 1913: 142; syn. n. Type Locality: Texas, USA. Type material: not found at NHMUK

##### Notes.

Zeller described *Enaemiapsammitis* from Texas in 1872. As early as 1874, Grote synonymized *L.psammitis* with *L.subfervens*; a decision followed subsequently by Barnes and McDunnough (1913, 1917). Heppner and Duckworth (1983) gave *L.psammitis* species status in their checklist without explanation. Perhaps, *L.psammitis* has persisted because phenotypes of *L.subfervens*, mostly from Oklahoma and Texas, with reduced forewing speckling were thought to represent a species independent from *L.subfervens*. Based on extensive barcode and phylogenetic data, multiple larval collections, and genitalic dissections, we see no evidence to suggest *L.psammitis* is a valid species. The original description of *L.psammitis* and type specimen in the NHMUK agree in detail with forms of *L.subfervens*, so we follow others and return the name to synonymy.

*Lacturarhodocentra* (*Miezarhodocentra*) was described from Texas by Meyrick in 1913. Shortly thereafter, it was designated as a synonym of *L.basistriga* by Barnes and McDunnough (1917) without explanation. Meyrick’s description which mentions “with a few scattered red [forewing] scales,” unambiguously places it as a form of *L.subfervens*. While the type of *L.rhodocentra* cannot be located at the Natural History Museum in London, we are not convinced that it no longer exists, because there are random pockets of Meyrick specimens spread throughout the large accumulation of “accessions” of unsorted microlepidoptera. Clarke (1955) did not include a depository for this species, suggesting the type was not present at the NHMUK at that time. Furthermore, it is unlikely that Meyrick would have had access to specimens of *L.basistriga*, *L.nalli*, or *L.rubritegula*, given that all three are geographically restricted to south Texas, and distant from major population centers or travel routes of the early 1900s. We have collected *Lactura* larvae and adults from various locales in eastern Texas, the likely source of the types for both names, examined the *Lactura* collections of Texas A&M University and the private collections of the late Edward Knudson (Houston) and James McDermott (Dallas area), checked barcodes for all US specimens, and see no evidence for the existence of a species that differs from the six treated species in this work. Like *L.psammitis*, we regard this name to be a synonym of *Lacturasubfervens*.

**Figures 15–22.**
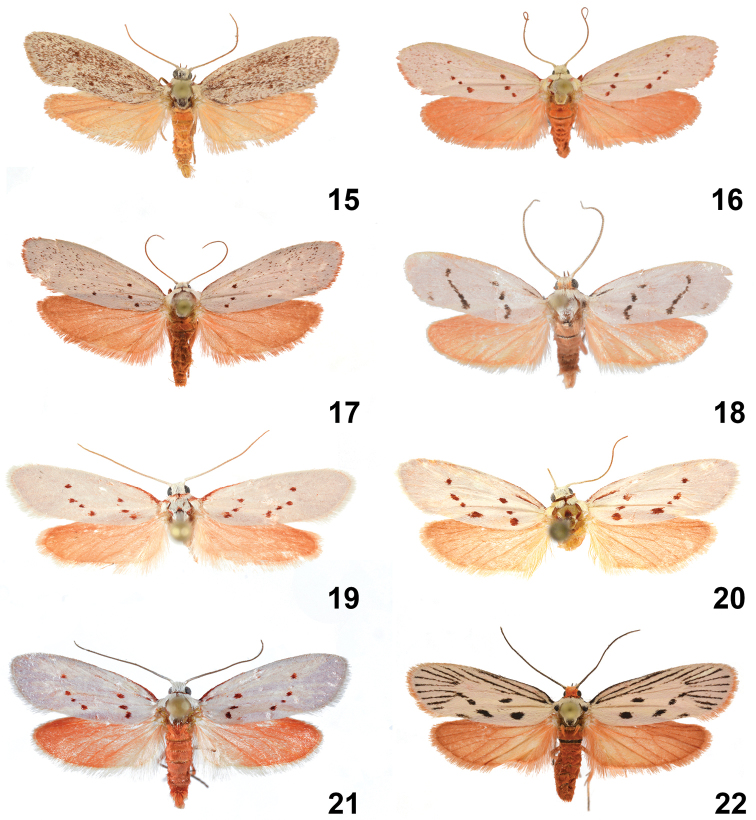
Adult *Lactura*. **15***Lacturasubfervens* (heavy maculation), TX: Kaufman Co., Becker, 18 February 2011, J. McDermott coll., CO1 Barcode DLW-000111 **16***Lacturasubfervens* (light maculation), TX: Harrison Co., Caddo Lake St. Pk., 14 June 1998, E. Knudson coll., CO1 Barcode DLW-000509 **17***Lacturasubfervenssapeloensis*, FL: Marion Co., Hopkins Prairie (29.275N, 81.692W), 18 March 2013, J. Vargo coll **18***Lacturaatrolinea*, TX: Cameron Co., Sabal Palm Grove Sanctuary, 16–17 November 1998, E. Knudson coll., genitalia slide #TAM-2017-015, CO1 Barcode DLW-000510 **19***Lacturarubritegula* [HOLOTYPE], TX: Kendall Co., Boerne, D. Cain Home (29°52'51"N, 98°36'51"W), 27 April 2015, David Wagner & Delmar Cain colls., genitalia slide #TAM-2017-002, CO1 Barcode DLW-000816 **20***Lacturabasistriga*, TX: Cameron Co., Sabal Palm Grove (25°51'9"N, 97°25'3.8"W), BBN15#27a, larva collected 25 April 2015, emerged 21 May 2015, Berry Nall coll., host: *Sideroxyloncelastrinum*, genitalia slide #TAM-2017-005 **21***Lacturanalli* [PARATYPE], TX: Starr Co., Falcon Heights (26.5585N, 99.1220W), 19 March 2018, Berry Nall coll **22***Lacturapupula*, FL: Nassau Co., Fort Clinch State Park, Ft. Clinch Fernandina Beach, DLW Lot: 2014D136, larva: 28 April 2014, emerged: 22 May 2014, host: *Sideroxlontenax*, Richard Owen coll.

**Figures 23–30.**
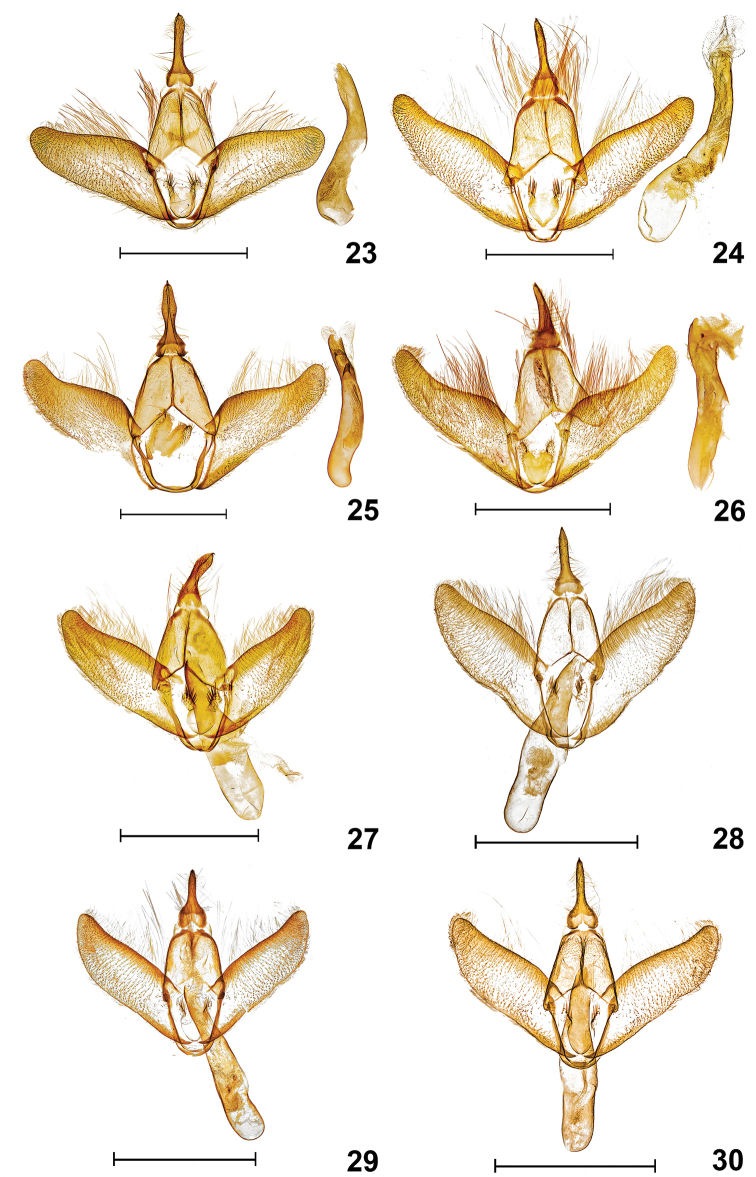
Male genitalia. **23***Lacturanalli* [PARATYPE], TX: Starr Co., Falcon Heights (26.5337N, 99.1059W), larva: 30 March 2014, pupated: 05 May 2014, emerged: 06 November 2014, host: *Sideroxyloncelestrinum*, Berry Nall coll., BBN14#06c, genitalia slide #TAM-2017-014, CO1 Barcode DLW-000486 **24***Lacturasubfervenssapeloensis* [PARATYPE], GA: McIntosh Co., Sapelo Island, Lighthouse Rd. Salt marsh edge habitat (31°23'25.7"N, 81°16'55"W), 11–12 March 2016, James Adams and Brian Scholtens colls., genitalia slide #TAM-2017-018, Voucher Code TAM0010 **25***Lacturaatrolinea*, TX: Cameron Co., Sabal Palm Grove Sanctuary, 16–17 November 1998, E. Knudson coll., genitalia slide #TAM-2017-015, CO1 Barcode DLW-000510 **26***Lacturapupula*, FL: Nassau Co., Fort Clinch St. Pk. Ferdinanda Beach, larva: 28 April 2014, emerged: 24 May 2014, host: *Sideroxylontenax*, Richard Owen coll., DLW Lot: 2014D136, Genitalia slide #TAM-2017-016, CO1 Barcode DLW-000282, Voucher Code TAM0009 **27***Lacturasubfervens*, TX: Uvalde Co., Neil’s Lodges, Rio Frio, larva: 17 April 2014, emerged: 16 April 2015, host: *Sideroxylonlanuginosum*, David L. Wagner coll., DLW Lot: 2014D45, genitalia slide #TAM-2017-019, Voucher Code TAM0007 **28***Lacturabasistriga*, TX: Hidalgo Co., Bentsen St. Pk., 30 April 1995, E. Knudson coll., genitalia slide #TAM-2017-003, CO1 Barcode DLW-000513 **29***Lacturarubritegula* [PARATYPE], TX: Kendall Co., Boerne (29°52'51"N, 98°36'50"W), 27 April 2015, David Wagner and Delmar Cain colls., genitalia slide #TAM-2017-004 **30***Lacturarubritegula* [HOLOTYPE], TX: Kendall Co., Boerne, D. Cain Home (29°52'51N, 98°36'51"W), 27 April 2015, David Wagner & Delmar Cain colls., genitalia slide #TAM-2017-002, CO1 Barcode DLW-000816. Scale bar: 1mm. Dissections and images prepared by Tony Thomas.

##### Diagnosis.

*Lacturasubfervens* adults can be immediately distinguished by the presence of scattered smoky red scales (although highly variable in density) over the forewing. The forewing lacks the basal subcostal red or black dash usually present in *L.atrolinea*, *L.basistriga*, and *L.nalli*. This species can also be separated from *L.basistriga*, *L.nalli*, and *L.rubritegula* by the absence of red scaling at the base of the patagium. Female genitalia differ from those of other *Lactura* in having 11 or 12 coils along the ductus bursae. The caterpillar is among the most distinct of the North America *Lactura*: it is the only species whose ground color is predominately white; seven pairs of pale stripes run the length of the body, giving the larva a frosted appearance. The darker stripes are due to the larva’s internal coloration showing through its transparent body wall; with the exception of the prothoracic shield, there is essentially no black pigmentation dorsally or laterally along the trunk.

##### Description adult

(Figs 15–17). Forewing length: 9–13 mm (n = 495). **Head.** Shiny, white decumbent scales over vertex and frons. Labial palpus slightly porrect to straight, brick red, subequal to diameter of eye. Antenna filiform, 2/3 length of forewing; shiny, white above, fuscous below. **Thorax.** Predominantly white. Patagium white; tegula with conspicuous basal band of red scales, similar to *L.rubritegula*. Medial mesothoracic red spot flanked posterolaterally by red ellipsoid to bar-like spots. Coxae, femora, and tibiae with red dorsal surface, and white or admixture of white- and red-scaled ventral surface; tarsi fuscous to red. **Forewing.** Mostly white, variable. Typically with seven crimson- to russet-red spots in antemedial and postmedial oblique series. Spotting sometimes reduced, but generally antemedial series with three spots and postmedial series with four spots. Varying degrees of scattered red to brown scales throughout. Some individuals with speckling so thick as to obscure antemedial and postmedial spots; other forms with only a few red or fuscous scales. Basal red scaling along costa narrows and then ends before antemedial spots. Fringe scales usually matching white forewing, although some individuals with red fringe. Underside light red with matching or slightly paler fringe scales. **Hindwing.** Uniformly light red above and below. **Abdomen.** Dorsum and sides brick red; venter rusty white. Ventral intersegmental hairpencils (with 40–60 androconial scales) inserted between A7 and A8. A second set of brushes between A6 and A7 was found in three preparations (Figs 33, 36). **Male Genitalia** (Figs 24, 27) (n = 6). Uncus strongly down-curved; medially constricted in basal third; distal part cylindrical and tapered (usually longer than congeners), terminating in reduced thorn-like apical spine. Valva elongate-oval, 2.5 × longer than wide, costa slightly concave along distal third; apex broadly rounded; lateral lobe of juxta with 15–20+ thickened spiniform setae. Vinculum narrow, U-shaped, subquadrangular. Aedeagus cylindrical, exceeding length of valva; base broadly rounded, gradually narrowing to apex; apex with concave oblique aperture; subapical thumb-like process absent. **Female genitalia** (Figs 42, 43) (n = 3). Papillae anales ca. 4 × longer than broad with dorsal sclerotized rim fused with posterior apophyses. Ductus bursae with 11 or 12 coils, posterior two coils more open; coil diameters more or less uniform with anteriormost coil slightly larger than others. Corpus bursae longer than broad with quadrate signa reduced; arms fused; lightly sclerotized in one preparation (USNM 76686). Corpus bursae without anterior accessory pouch.

##### Description of living final instar

(Fig. 45). Appearing frosty or pale green or exceptionally yellowish; seven pairs of whitish stripes run length of body; without black pigment dorsally or laterally. D2 on elevated yellow verrucae connected by thick yellow to white subdorsal stripe. Prothoracic shield well differentiated, medially divided, with much less black dorsal pigmentation relative to congeners. Head brown, partially retracted into prothorax.

##### Distribution and biology.

*Lacturasubfervens* is found in woodlands, bottomlands, and thickets of central Texas northward to southeast Kansas, Missouri, and Illinois, and east through Gulf States to coastal Georgia (Fig. 53). Its range is largely sympatric with *L.pupula*, with both species mirroring the distribution of one of their shared host plants, *Sideroxylonlanuginosum*. *Lacturasubfervens* is not found in southern Florida, unlike *L.pupula*. The species is essentially univoltine, typically flying very early in season (January to April) (Figs 58, 59), when *Sideroxylon* is producing new leaves, although there are smaller, facultative broods across the southern portions of its range, with captures into June and occasionally into the fall. We suspect that larvae from these adults commonly fail due to lack of appropriate foliage. From at least the Dallas area west into the Hill Country region of Texas, *L.subfervens* can reach larval densities high enough to severely damage or completely defoliate *Sideroxylonlanuginosum*.

##### Remarks.

For decades, less speckled forms of *L.subfervens* have been misidentified as *L.basistriga* in collections, literature (Heppner 2003), and on commonly used internet sites, e.g., BugGuide, iNaturalist, and Moth Photographers Group. Much of this confusion was avoidable and speaks to the merit (and necessity) of revisiting original descriptions and examining types. Barnes and McDunnough’s (1913) original description of *L.basistriga* provides clear diagnostic characters that unambiguously distinguished the adult from *L.subfervens*: “lacking the brown streaks on the forewings and in having a red basal streak below the costa,” although the latter character is sometimes absent.

#### Lactura subfervens sapeloensis

Taxon classificationAnimaliaLepidopteraLacturidae

Matson & Wagner ssp. n.

http://zoobank.org/A187618D-1434-4FA4-BFDC-1F2C9400EC65

Figs 17 , 24 , 33 , 36 , 43 , 59 , 63 , Table 1

##### Notes.

*Lacturasubfervens* collections from central and coastal Florida, and coastal Georgia show modest genetic differentiation (pairwise distance ~0.02) (Fig. 63) from *L.subfervens* populations found further west, i.e., from Alabama to Texas north into the Great Plains. We describe this geographic segregate as a new subspecies: *Lacturasubfervenssapeloensis*. It is possible that the phylogeographic structure arose when central Florida was separated from the mainland during interglacial rises in sea level (Neill 1957). In early May of 2017, Brian Scholtens and James Adams collected females of *Lacturasubfervenssapeloenisis* from Sapelo Island, Georgia. While the neonates established on *Sideroxyloncelastrinum*, mortality was high because there was little new growth on the plants: most larvae failed in the penultimate instar with only one making it into the final instar. The single final instar larva displayed a darker green dorsum (no photograph available) than the *L.subfervens* that we have imaged or reared from Texas. Given the large role that larval differences played in this work, it would be valuable if wild larvae from Sapelo Island, Georgia, or Florida, could be collected and evaluated to assess the larval features of these southeastern populations. As noted above, many have long misidentified some forms of this moth as *L.basistriga* (see *L.subfervens* Remarks).

**Figures 31–36.**
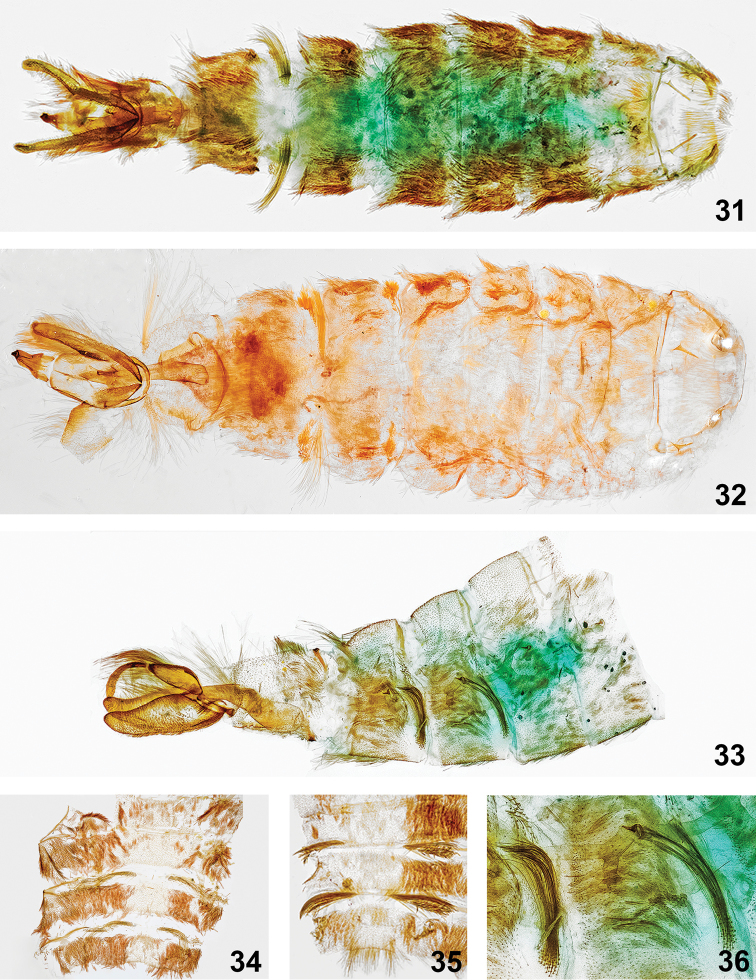
Male abdomens with scent-emitting androconial structures. **31***Lacturapupula*, FL: Nassau Co., Fort Clinch St. Pk. Ferdinanda Beach, DLW Lot: 2014D136, larva: 28 April 2014, emerged: 24 May 2014, host: *Sideroxylontenax*, Richard Owen coll., Genitalia slide # TAM-2017-016, CO1 Barcode DLW-000282, Voucher Code TAM0009 **32***Lacturaatrolinea*, TX: Cameron Co., Sabal Palm Sanctuary (25°51'3"N, 97°25'1"W), ex-ova: female 25 November 2014, DLW Lot: 2014L121b, emerged: 17 February 2015, host: *Sideroxyloncelastrinum*, genitalia slide #TAM-2017-006, Voucher Code TAM0008. **33***Lacturasubfervenssapeloensis* [PARATYPE], GA: McIntosh Co., Sapelo Island, Lighthouse Rd. salt marsh edge habitat (31°23'25.7"N, 81°16'55"W), 11–12 March 2016, James Adams and Brian Scholtens coll., genitalia slide #TAM-2017-018, Voucher Code TAM0010 **34***Lacturanalli* [PARATYPE], TX: Starr Co., Falcon Heights (26.5337N, 99.1059W), larva: 30 March 2014, pupated: 05 May 2014, emerged: 06 November 2014, host: *Sideroxyloncelastrinum*, Berry Nall coll., BBN14#06c, genitalia slide #TAM-2017-014, CO1 Barcode DLW-000486 **35***Lacturaatrolinea*, TX: Cameron Co., Audubon Sabal Palm Grove Sanctuary, 16–17 November 1998, E. Knudson coll., genitalia slide # TAM-2017-015, CO1 Barcode DLW-000510 **36***Lacturasubfervenssapeloensis*, see 33 above. Dissections and images prepared by Tony Thomas.

**Figures 37–43.**
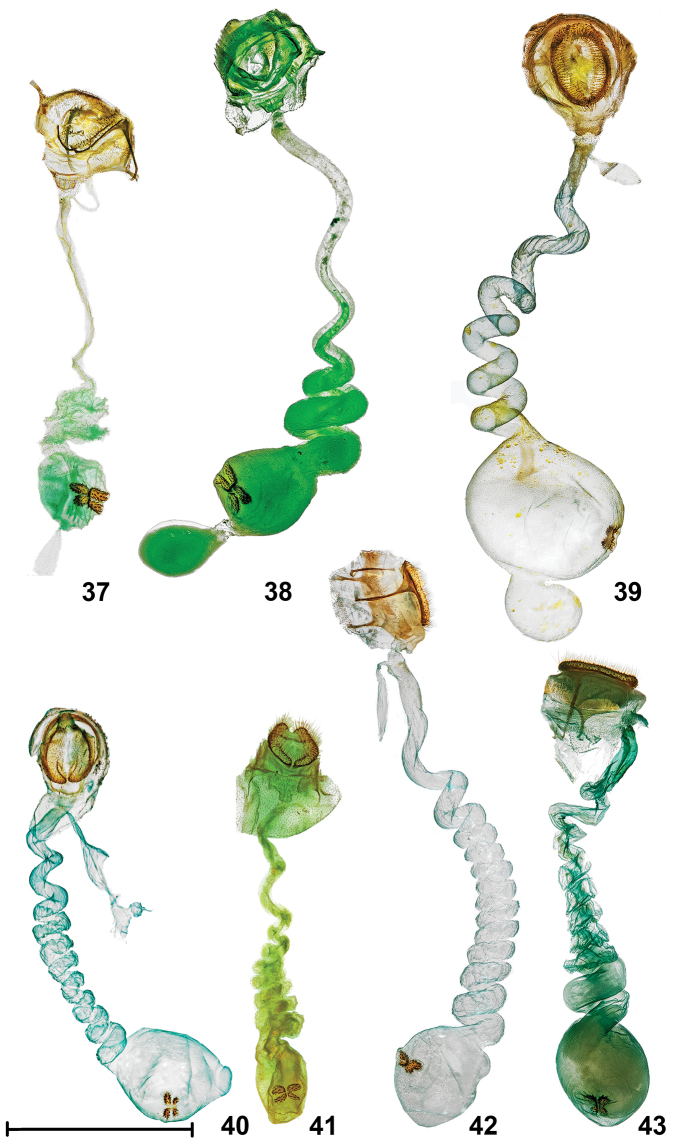
Female genitalia. **37***Lacturabasistriga*, TX: Cameron Co., Sabal Palm Sanctuary (25°51'9"N, 97°25'3.8"W), larva: 25 April 2015, pupated: 29 April 2015, emerged: 21 May 2015, host: *Sideroxyloncelestrinum*, Berry Nall coll., BBN15#27a, genitalia slide #TAM-2017-005, Voucher Code TAM0005 **38***Lacturanalli* [PARATYPE], TX: Starr Co., Falcon Heights (26.5337N, 99.1059W), genitalia slide #TAM-2017-013, CO1 Barcode DLW-000569 **39***Lacturaatrolinea*, TX: Cameron Co., Sabal Palm Sanctuary (25°51'3"N, 97°25'1"W), 25 November 2014, David L. Wagner coll., genitalia slide #TAM-2017-007 **40***Lacturapupula*, FL: Duval Co., Little Talbot State Park, 24 March 2007, B.D. Williams coll., genitalia slide #TAM-2017-011 **41***Lacturarubritegula* [PARATYPE], TX: Kendall Co., Boerne (29°52'51"N, 98°36'51"W), 27 April 2015, David L. Wagner coll., ♀ DLW 2015D60.2b, genitalia slide #TAM-2017-001 **42***Lacturasubfervens*, TX: Comal Co., New Braunfells off Huaco Sprgs, Loop Road, 24 March 1995, genitalia slide #TAM-2017-009 **43***Lacturasubfervenssapeloensis* [PARATYPE], GA: McIntosh Co., Sapelo Island, Lighthouse Rd. salt marsh edge habitat (31°23'25.7"N, 81°16'55"W), 11–12 March 2016, James Adams and Brian Scholtens colls., genitalia slide #TAM-2017-017, CO1 Barcode DLW-000770, Voucher Code TAM0003. Scale bar: 2 mm. Dissections and images prepared by Tony Thomas.

##### Diagnosis.

*Lacturasubfervenssapeloensis* is most easily identified by its Florida/Georgia distribution. We have not found consistent diagnostic characters that will distinguish it from *L.subfervens* in adult pattern or male genitalia. Female genitalia do show modest differentiation: there are 12 or13 coils in the ductus bursae of *L.subfervens* and nine or ten coils in our preparations of *L.subfervenssapeloensis*.

##### Etymology.

We derived this trinomen from Sapelo Island, Georgia, where the moth is particularly common, and from which most of the paratype series was collected.

##### Distribution and biology.

*Lacturasubfervenssapeloensis* is found in coastal strand communities, mesic woodlands, thickets, flatwoods, scrublands, and edges of wetlands from central Florida, north into southeast Georgia (Fig. 53). The moth has been collected south to the Archbold Biological Station in Highlands Co., Florida, although recent collections from this area are modest in number. Its range overlaps with southeastern populations of *L.pupula*. We suspect that a primary hostplant for the larva will prove to be *Sideroxylontenax* (a dominant *Sideroxylon* in Florida) instead of the more widely distributed *Sideroxylonlanuginosum*, which is used by *L.pupula*, *L.rubritegula*, and *L.subfervens* to the west. As far as known, there is one principle spring brood (Fig. 59).

##### Type material.

**Holotype male**, dry pinned, GA: McIntosh Co., Sapelo Island, Lighthouse Rd., salt marsh edge habitat (31°23'25.7"N, 81°16'55"W), 11–12 March 2016, light trap, James Adams & Brian Scholtens coll., genitalia slide #TAM–2017– 017, CO1 Barcode DLW–000570, Voucher Code TAM0003, Deposited at USNM, Washington D.C., USA. **Paratypes adults** (14♂, 14♀): GA: McIntosh Co., Sapelo Island, Beach Rd nr. greenhouse (31.397436N, 81.274092W), 21–22 April 2017, MV light, Tanner A. Matson coll. (1♀) (FMNH); GA: McIntosh Co., Sapelo Island, Lighthouse Rd., salt marsh edge habitat (31°23'25.7"N, 81°16'55"W), 11–12 March 2016, light trap, James Adams & Brian Scholtens coll., genitalia slide #TAM–2017– 018, Voucher Code TAM0010 (1♂) (UCMS); GA: Camden Co., Little Cumberland Island (30°58'N, 81°25'W), 15–19 March 1997, W. E. Steiner et al. coll., CO1 Barcode LNAUV110-16, LNAUV174-16, Voucher Code USNMENT 01237375, 01237311 (1♂, 2♀) (USNM); FL: [Highlands Co.], Lake Placid, Archbold Bio. Sta., 27 March 1959, Ronald W. Hodges coll., genitalia slide 827, CO1 Barcode LNAUS279-12, Voucher Code USNMENT 00831263 (1♀) (USNM); FL: Marion Co., Hopkins Prairie (29.275N, 81.692W), 18 March 2013, Jim Vargo coll. (2♀) (FMNH); FL: Martin Co., Hopkins Prairie (27.00N, 80.142W), 05 February 2014, Jim Vargo coll. (1♂) (UCMS); GA: McIntosh Co., Sapelo Island, Lighthouse (31.391500N, 81.285703W), 17 July 2015, UV light trap, Lance Durden coll., BGS collection #BGSGA05096 (1♂) (CC); GA: McIntosh Co., Sapelo Island, Nannygoat Beach dunes (31.390N, 81.265W), 10 May 2012, UV light trap, Lance Durden coll., BGS collection #BGSGA02307 (1♂) (CC); GA: McIntosh Co., Sapelo Island, Old Beach Rd. (31.4064N, 81.2592W), 21 May 2014, UV light trap, John Hyatt coll., BGS collection #BGSGA02318 (1♂) (CC); GA: McIntosh Co., Sapelo Island, Lighthouse Rd., salt marsh edge habitat (31°23'25.7"N, 81°16'55"W), 11–12 March 2016, light trap, James Adams and Brian Scholtens coll., BGS collection #BGSGA02636, #BGSGA02637, #BGSGA02638, #BGSGA02639 (1♂, 3♀) (CC); GA: McIntosh Co., Sapelo Island, trailer opening (31.399N, 81.281W), 10 Mar 2016, MV light, Brian Scholtens coll., BGS collection #BGSGA02640 (1♀) (CC); GA, McIntosh Co., Sapelo Island, dune ca. bridge (31.3917N, 81.2685W), 11 March 2016, UV light trap, Brian Scholtens coll., BGS collection #BGSGA02641 (1♂) (CC); GA, McIntosh Co., Sapelo Island, Nannygoat Beach dunes (31.390N, 81.265W), 10 Mar 2016, UV light trap, Brian Scholtens coll., BGS collection #BGSGA02642 (1♂) (CC); GA: McIntosh Co., Sapelo Island, Lighthouse Rd., salt marsh edge habitat (31°23'25.7"N, 81°16'55"W), 11–12 March 2016, light trap, James Adams & Brian Scholtens coll., (3♂, 2♀) (JKA); GA: McIntosh Co., Sapelo Island, beach habitat, end of Beach Rd. light trap (31°23'26.5"N, 81°15'54.5"W), 10–12 March 2016, James K. Adams & Brian Scholtens coll., (1♂) (JKA); same locality, 8–10 March 2017, James K. Adams & Brian Scholtens coll. (1♂, 1♀) (JKA); GA: McIntosh Co., Sapelo Island, near UGA dorms (31°23'54"N, 81°16'51"W), 12 March 2016, at light trap, James Adams coll. (1♀)(JKA).

#### Lactura atrolinea

Taxon classificationAnimaliaLepidopteraLacturidae

(Barnes & McDunnough, 1913)

Figs 18 , 25 , 32 , 35 , 39 , 47 , 53 , 57 , 63 , Table 1

Mieza atrolinea Barnes and McDunnough, 1913: 142. Type locality: San Benito, Texas, USA. Type material: USNM

##### Diagnosis.

*Lacturaatrolinea* is easily distinguished by its larger size and nearly continuous series of black antemedial and postmedial spots. The tegula is black apically as are the spots over the thorax; the forewing has a thin black subcostal dash. In females the ductus bursae has five or six coils anteriorly. The larva displays attractive, metallic blue, dorsal warts that unmistakably separate this species from its co-occurring Texas congeners.

**Figures 44–49.**
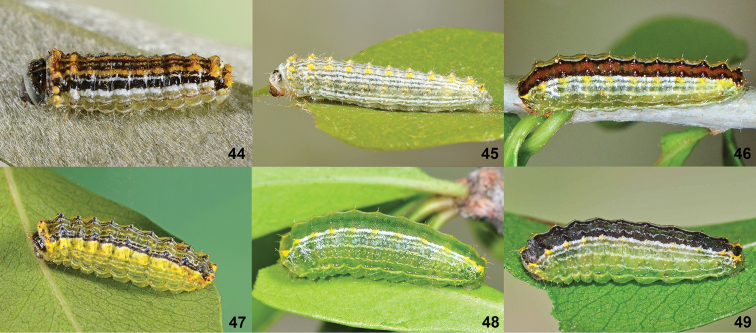
*Lactura* last instars. **44***Lacturapupula***45***Lacturasubfervens***46***Lacturarubritegula***47***Lacturaatrolinea***48***Lacturanalli***49***Lacturabasistriga*.

**Figures 50, 51.**
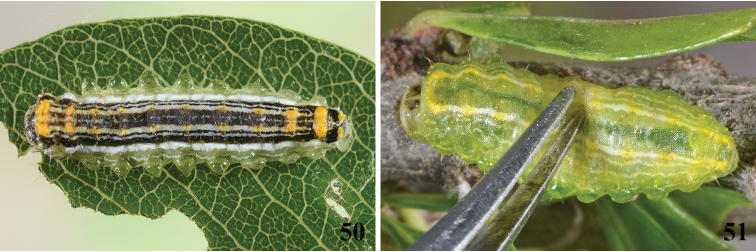
When threatened, *Lactura* caterpillars evert transparent, balloon-like vesicles from the side of their body that secrete a sticky, mucilaginous fluid. **50***Lacturapupula* larval defense response **51***Lacturanalli* larval defense response.

**Figures 52, 53.**
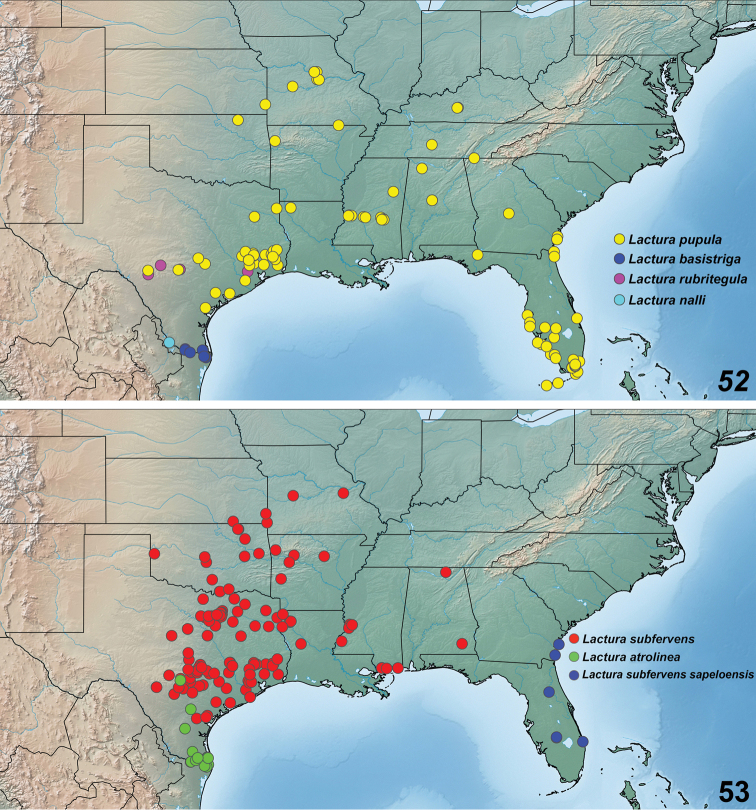
Geographic distribution of *Lactura* north of Mexico. Single dots may represent >1 individuals. **52***Lacturapupula* (n = 231), *Lacturabasistriga* (n = 93), *Lacturarubritegula* (n = 13), and *Lacturanalli* (n = 11) **53***Lacturasubfervens* (n = 472), *Lacturaatrolinea* (n = 138), and *Lacturasubfervenssapeloensis* (n = 20).

##### Description adult.

(Fig. 18) Forewing length: 9–13 mm (n = 138). **Head.** Vertex white with paired, addorsal, salmon scale patches; frons with shiny white decumbent scales. Labial palpus slightly porrect, brick red at base and black at terminus, subequal to diameter of eye. Antenna filiform, 0.6 length of forewing; shiny, white decumbent scales over scape and basal 2/5, transitioning to fuscous with scattered whitish scales; distal 1/5 brick red. **Thorax.** Predominantly white. Patagium white with a few red basal scales. Tegula with small ventral black basal patch; white medially and shiny black apically. Prothorax with lateral pair of black spots, but these sometimes obscured by patagium. Coxa and femur with red dorsal surface and light red to pale white outer and ventral surfaces; procoxa with basal mixture of red and black scales; pro- and mesotibia and pro- and mesotarsus black or fuscous dorsally and fuscous red ventrally; metatibia light red; metatarsus fuscous red. **Forewing.** Pearly white, with oblique series of black antemedial and postmedial spots. Spots nearly contiguous to fused. Antemedial row with three spots; lower spot largest and rendering row convex with respect to base of forewing. Postmedial series with heavy black dash approximately 1/2 width of forewing (continuous but narrowing anteriorly); smaller spot above. Inconspicuous black spot near tornus. Black, basal, subcostal dash similar in size and placement to red subcostal dash in *L.basistriga*. Basal black scaling along costa narrows and ends before antemedial spots. Fringe scales white. Underside red. **Hindwing.** Uniformly light red to orange with white fringe scales. Underside of hindwing paler than those of underside of forewing. **Abdomen.** Dorsum and sides brick red; venter rusty white. Two pairs of subventral intersegmental hairpencils (with 40–60 androconial scales) inserted between A6 and A7, and A7 and A8 (Figs 32, 35). Roseate cluster of elliptic scales, 5 × longer than broad, located anterior to spiracle in intersegmental folds, much more pronounced than in other Texas congeners. Hairpencils and roseate scale clusters more extensive between A7 and A8 than between A6 and A7. **Male Genitalia** (Fig. 25) (n = 6). Uncus more robust than congeners, strongly down-curved and tapering, strong medial constriction in basal third, ending in short thorn-like apical spine. Valva elongate-oval, 2× longer than wide, costa concave along distal third; broadly rounded at apex; outer margin with shorter, thicker scales; lateral lobe of juxta similar to *L.pupula*, but more sclerotized and prominent, with 20–30+ spiniform setae. Vinculum narrow, U-shaped, subquadrangular. Aedeagus cylindrical, exceeding length of valva; base broadly rounded; apex with gaping concave aperture and subapical thumb-like process twice as long as wide. **Female genitalia** (Fig. 39) (n = 4). Papillae anales ca. 4 × longer than broad with dorsal sclerotized rim fused with posterior apophyses. Ductus bursae with five or six coils; coils more open than other treated species; posterior two coils more open and extended, with corrugated surface; remaining (anterior) coils tightly bound with little to no surface corrugation. Corpus bursae longer than broad; four lobes of signa large with dentate interior projections; teeth widely spaced (similar to those of *L.basistriga*). Corpus bursae with anterior accessory pouch with broad opening.

##### Description of living final instar.

(Fig. 47) Ground color yellow to lime green. D1 and D2 setae borne from metallic blue warts. Middorsum with yellow middorsal stripe; green addorsal stripe running through D1 wart; the latter bound laterally by white pinstripe. Doubled, black dorsal/subdorsal stripe running through larger D2 wart. Supraspiracular area broadly yellow, transitioning to green through spiracular region. Light blue SD1 pinacula positioned directly above spiracles on A1–A8; these connected by thin, wavy, often broken lateral yellow line. A thicker, continuous, stripe runs just below the tan-yellow spiracles.

##### Distribution and biology.

*Lacturaatrolinea* inhabits the mesic woodlands, coastal scrub, and palm forests of south Texas, southward into Mexico. Breeding populations are known from coastal areas of Texas from Fort Bend to Cameron counties (Fig. 53). What may represent strays have also been taken from the south-central Texas counties of Medina, Live Oak, and Duval. Multiple generations each year with peak activity tied to early spring (March to May) and the fall rainy periods (September into November) (Fig. 57). Larvae feed on saffron plum (*Sideroxyloncelastrinum*); caterpillars and adults are sometimes common in the Sabal Palm Sanctuary near Brownsville, Texas.

#### Lactura basistriga

Taxon classificationAnimaliaLepidopteraLacturidae

(Barnes & McDunnough, 1913)

Figs 20 , 28 , 37 , 49 , 52 , 61 , 63 , Table 1

Mieza basistriga Barnes and McDunnough, 1913: 142. Type locality: San Benito, Texas, USA. Type material: USNM

##### Diagnosis.

*Lacturabasistriga* can be distinguished from *L.rubritegula* by the absence of dorsal red scales on the tegula. Most forms exhibit a basal, red, subcostal dash on the forewing, and all individuals lack the scattered flecking of red or brown scales characteristic of *L.subfervens*. Many, but not all individuals can be distinguished from *L.rubritegula* by a reduced upper postmedial spot and the convex arcing of the three lower postmedial spots, due to a more basal placement on the lowermost spot. Adults are exceedingly close to and often indistinguishable from those of *L.nalli*; see comments under that species. With the exception of *L.nalli* and *L.atrolinea*, female genitalia differ from other *Lactura* in having four or five coils in ductus bursae. Larvae with a dark dorsum that ranges from smoky gray-green to nearly black, and without the metallic blue dorsal pinacula of *L.atrolinea*; larvae of *L.nalli* are lime green above and lack any hint of white striping or black fill between the subdorsal stripes that are present in *L.basistriga*.

**Figures 54–59.**
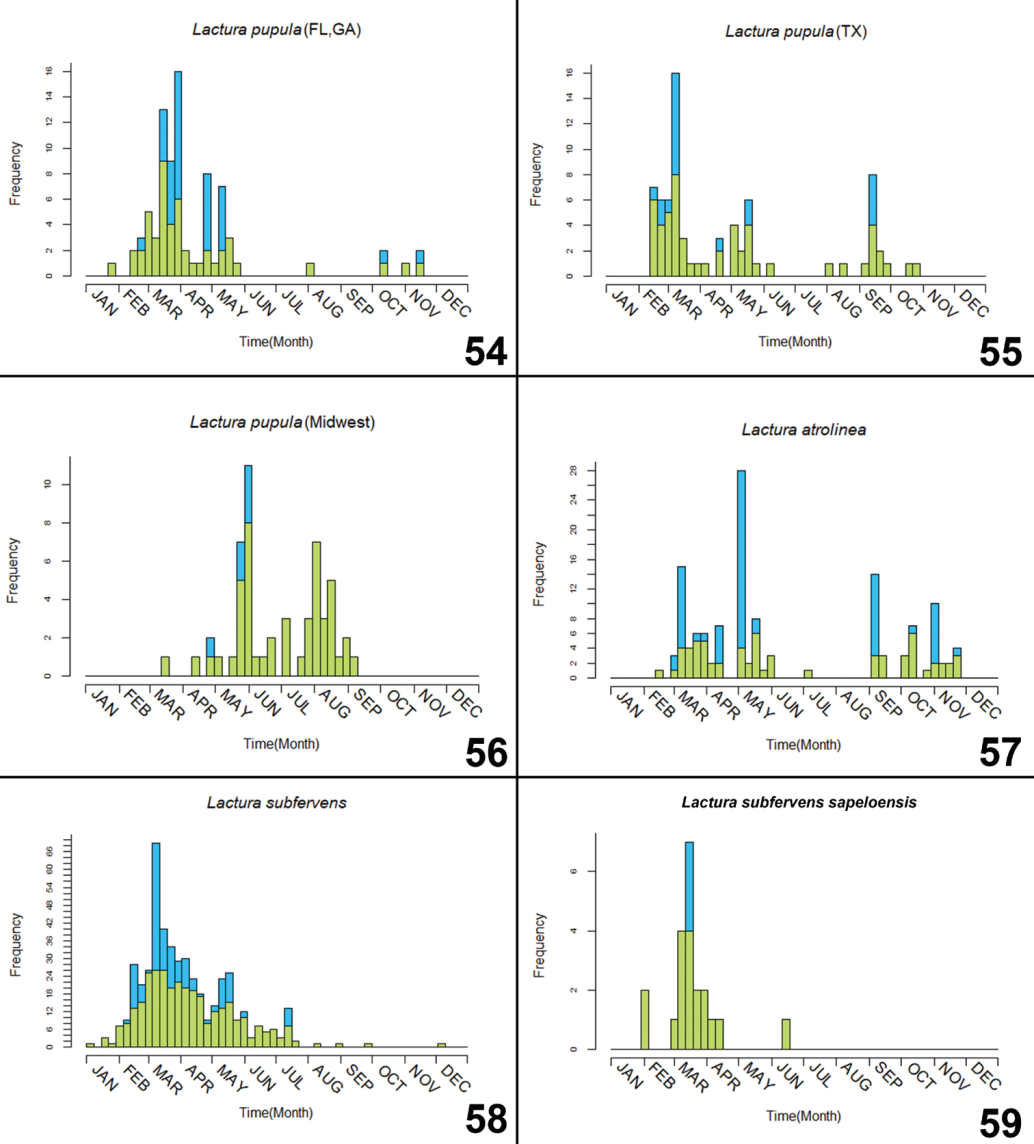
Phenology of North American *Lactura*, (blue square) all specimen records, (green square) records representing a unique collecting event (multiple individuals from the same collecting event represent a single data point). **54***L.pupula* from Florida/Georgia (n = 82) **55***L.pupula* from Texas (n = 74) **56***L.pupula* from Midwest, U.S. (n = 54) **57***L.atrolinea* (n = 138) **58***L.subfervens* (n = 474) **59**L.subfervenssapeloensis (n = 21).

**Figures 60–62.**
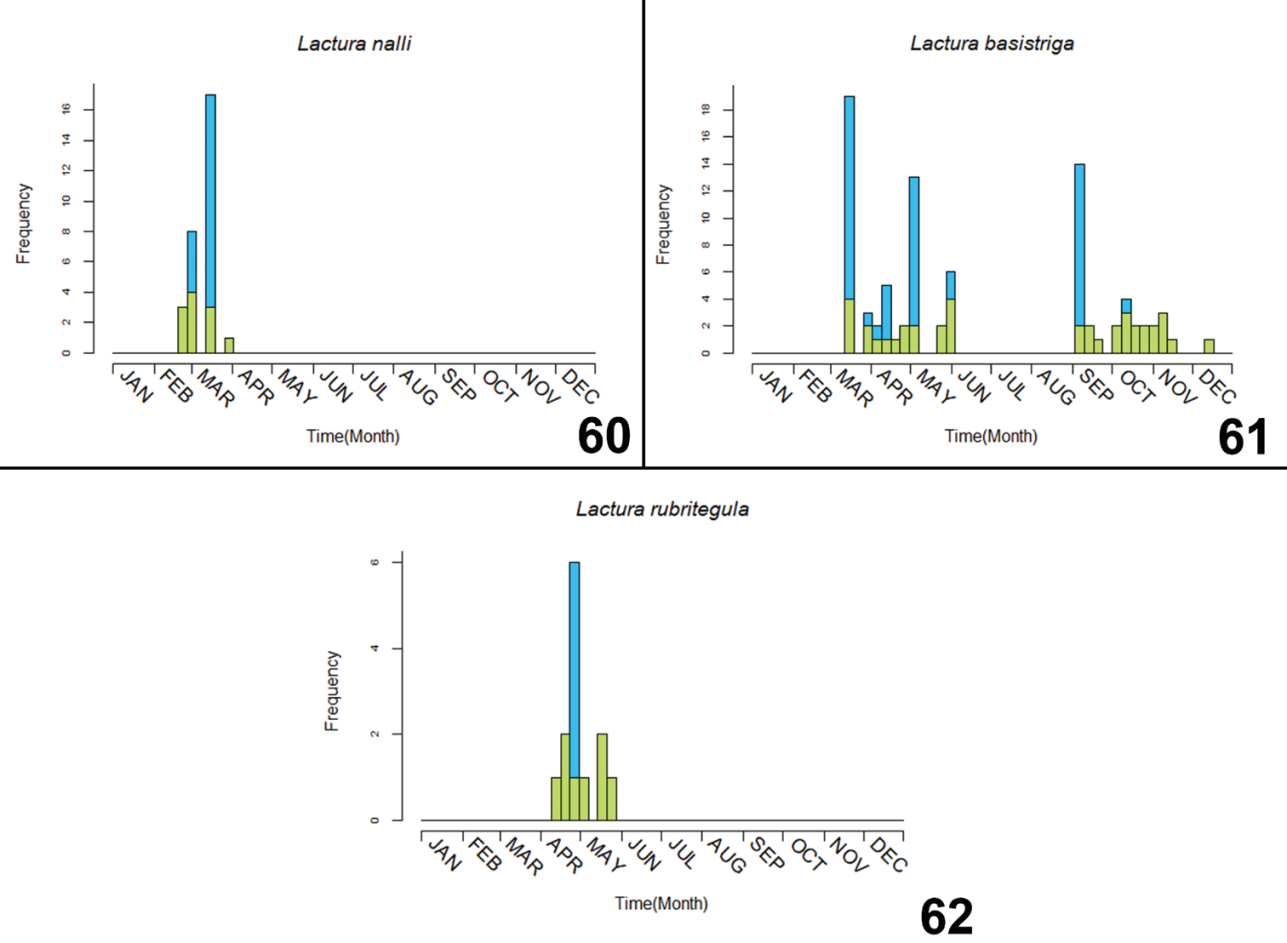
Phenology of North American *Lactura*, (blue square) all specimen records, (green square) records representing a unique collecting event (multiple individuals from the same collecting event represent a single data point). **60***L.nalli* (n = 29) **61***L.basistriga* (n = 87) **62***L.rubritegula* (n = 13).

##### Description adult.

(Fig. 20) Forewing length: 9–11 mm (n = 97). **Head.** Shiny, white decumbent scales over vertex and frons. Labial palpi fuscous red, straight or slightly porrect. Antenna filiform, 0.6 length of forewing; shiny, white decumbent scales over scape and basal 1/3, transitioning to shiny, orange to fuscous orange scales; fuscous beneath. **Thorax.** Patagium mostly white; red basally, most apparent around perimeter of eye. Tegula white with small, ventral, red basal patch. Subtriangular, medial, mesothoracic red spot flanked posterolaterally by ellipsoid to bar-like red spots. Coxa and femur with reddish mesal surface, distal and ventral surfaces with admixture of both white and red scaling; tibia given more to white scaling; protarsus mostly red, meso- and metatarsus given to more white scaling. **Forewing.** Pearly white, with seven blood- to mahogany-red spots in oblique antemedial and postmedial series; without scattered dark scales (of *L.subfervens*). Antemedial row with three spots; lower and upper spots often larger than middle spot; postmedial series with four spots: uppermost usually smaller or subequal to that below it; lower three forming straight line or, more commonly, a convex arc facing the termen. (These same three spots often form a concave arc open to termen in *L.rubritegula* due to a more distal placement of the lowermost spot.) Basal subcostal red dash from which the species derives its specific epithet, typically ending before the uppermost antemedial spot; subcostal dash always present in males, but its development (length and thickness varying among individuals); basal red subcostal dash frequently absent in females. Basal red scaling along costa narrows and ends before antemedial spots. Underside red with white fringe scales. **Hindwing.** Uniformly light orange-red above; light orange-red below, becoming paler along inner margin. **Abdomen.** Dorsum and sides brick red; venter rusty white. Two pairs of subventral intersegmental hairpencils (with 40–60 androconial scales) inserted between A6 and A7, and A7 and A8. **Male Genitalia** (Fig. 28) (n = 3). Male genitalic characters overlap with those of *L.nalli* and *L.rubritegula*. **Female genitalia** (Fig. 37) (n = 2). Papillae anales ca. 4 × longer than broad with dorsal sclerotized rim anastomosed with posterior apophyses. Ductus bursae with four or five coils, posterior half sublinear, transitioning to strongly coiled anterior half; diameter of coils increasing to corpus bursae, anteriormost coil 3 × greater in diameter than posteriormost coil (subequal to width of corpus bursae). Corpus bursae longer than broad; signa consisting of four, large hemispherical lobes with dentate interior projections; teeth widely spaced (similar to *L.atrolinea*). Corpus bursae with anterior accessory pouch narrowed at its opening.

##### Description of living final instar.

(Fig. 49) Ground color glossy yellow, pale, or smoky green with variable black coloration over dorsum interrupted by wavy, white addorsal stripe. D2 seta borne from slightly elevated yellow wart connected by thick yellow to white subdorsal stripe; yellow warts less evident through midabdominal segments. Enlarged dorsal verrucae on A8 (bearing D1, D2, and SD1 setae), usually yellow or yellow-orange and black over mesal surface. Two pale, wavy supraspiracular stripes extending from T1–A8.

##### Distribution and biology.

*Lacturabasistriga* inhabits the woodlands, thickets, palm forests, and scrublands of the lower Rio Grande Valley; presumably the core of its range is in Mexico. Breeding populations are known from the extreme southern Texas counties of Hidalgo and Cameron (Fig. 52). This species is sympatric with *L.atrolinea* in the United States; the moths are active at the same time of year and both feed on new foliage of saffron plum (*Sideroxyloncelastrinum*). Peak activity is tied to early spring (March to May) and the fall rainy seasons (September into November) (Fig. 61).

#### Lactura rubritegula

Taxon classificationAnimaliaLepidopteraLacturidae

Matson & Wagner, 2017

Figs 1 , 19 , 29 , 30 , 41 , 46 , 52 , 62 , 63 , Table 1

Lactura rubritegula Matson & Wagner, 2017: 141. Type locality: Kendall Co., Texas, USA. Type material: USNM, UCMS, TAMUIC

##### Diagnosis.

*Lacturarubritegula* can be distinguished from its relative *L.basistriga* by the prominence of red dorsal scales on the tegula. It lacks the red subcostal dash that can be found in most forms of *L.basistriga* and *L.nalli*, and the scattered flecking of red or brown scales characteristic of *L.subfervens*. Many, but not all, individuals can be distinguished by the basal displacement of the lowermost antemedial spot, somewhat enlarged upper postmedial spot, and the concave arc (open to termen) of the three lower postmedial spots. Female genitalia differ from other *Lactura* in this treatment in having six or seven coils in the ductus bursae. Unique to the larva of this species is the rusty brown dorsum.

**Figure 63.**
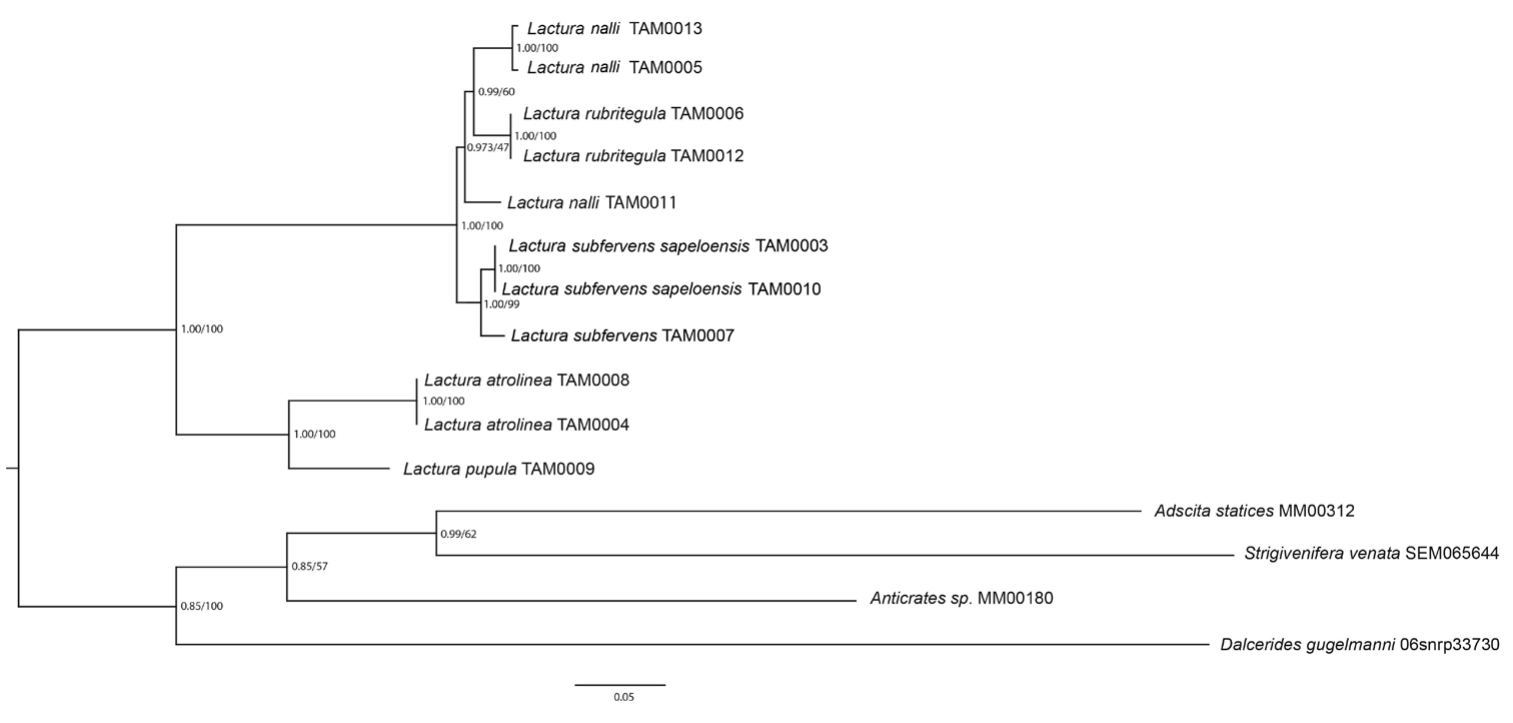
Likelihood tree for *Lactura* found north of Mexico with four outgroup zygaenoids based on nine gene regions – see Methods. Bayesian posterior probabilities (left) and bootstrap support values (right) for internal branches.

##### Description adult.

(Figs 1, 19) Forewing length: 9–11 mm (n = 25). **Head.** Shiny, white decumbent scales over vertex and frons; lower frons sometimes with scattered pink scales. Labial palpus slightly porrect to straight, brick red, subequal to diameter of eye. Antenna filiform, 0.6 length of forewing; shiny, white decumbent scales over scape and basal 2/5, transitioning to admixture of white and red scales; distal 1/5 brick red. **Thorax.** Predominantly white. Patagium mostly white, but red basally. Tegula with conspicuous basal and medial red scales, similar to *L.subfervens*. Medial mesothoracic red spot flanked posterolaterally by ellipsoid to bar-like red spots. Coxa and femur with reddish mesal and dorsal surfaces, distal and ventral surfaces mostly white with mixture of both white and red scaling; tibia given more to white scaling; protarsus mostly red, meso- and metatarsus given to more white scaling. **Forewing.** Pearly white, with seven blood- to mahogany-red spots in oblique antemedial and postmedial series; without scattered dark scales (of *L.subfervens*). Antemedial row with three spots; lower spot usually displaced basally and often smaller than middle spot; postmedial series with four spots: uppermost usually larger or subequal to that below it; lower three forming straight line or (more commonly) slightly concave arc open to termen. (These same three spots often form convex arc in *L.basistriga* and *L.nalli* due to basal displacement of lowermost spot.) Basal red scaling along costa narrows and ends before antemedial spots. Underside red with white fringe scales. **Hindwing.** Uniformly light red, above and below, with elongate white fringe scales. **Abdomen.** Dorsum and sides brick-red; venter rusty white. Two pairs of subventral intersegmental hairpencils (with 40–60 androconial scales) inserted between A6 and A7, and A7 and A8. **Male Genitalia** (Figs 29, 30) (n = 2). Uncus strongly down-curved; medially constricted in basal third; distal part cylindrical and tapered, terminating in thorn-like apical spine. Valva elongate-oval, 2.5 × longer than wide, costa concave at distal third; apex broadly rounded; lateral lobe of juxta with 10–20+ thickened spiniform setae. Vinculum narrow, U-shaped, subquadrangular. Aedeagus exceeding length of valva; thickest at midlength; base broadly rounded; apex ca. half width of middle section, ending in concave oblique aperture, subapical thumb-like process absent. **Female genitalia** (Fig. 41) (n = 2). Papillae anales ca. 4 × longer than broad with dorsal sclerotized rim fused with posterior apophyses. Ductus bursae with 6–8 coils; coil diameter gradually increasing to corpus bursae; anteriormost coil 2 × diameter of posteriormost coil, and subequal to diameter of bursa. Corpus bursae longer than broad with large four-lobed signa; without anterior accessory pouch.

##### Description of living final instar.

(Fig. 46) Glossy pale green with broad cinnamon-brown middorsal stripe outwardly edged with black that runs through the D1 pinacula; white dorsal/subdorsal stripe running through yellow D2 warts; two, wavy-edged, pale supraspiracular stripes, subequal in width to subdorsal stripe, that extend from T1–A8. Larger primary setae borne from minute white spots (~pinacula). Dorsum with vague, black, transverse intersegmental lines. White D1 pinacula borne from apex of otherwise black warts. D2 seta from white pinaculum at apex of yellow wart with yellow extending down to SD seta. Thin, vague, wavy, pale spiracular stripe immediately ventral to light-orange spiracles, as well as single, white, straight-edged subventral stripe equal in width to supraspiracular stripes.

##### Distribution and biology.

*Lacturarubritegula* is known from the Hill Country around San Antonio, Texas, westward to Edwards and Uvalde counties, but its range remains unclarified due to previous taxonomic confusion with *L.basistriga* and other *Lactura* (Fig. 52). Its range likely extends into Mexico. Delmar Cain has found larvae feeding on *Sideroxylonlanuginosum* (this is the only *Sideroxylon* that occurs at the three known localities for the species). Peak flight of *L.rubritegula* appears to be tied to spring rains and the availability of new foliage. The moth begins flying, late for a *Lactura*, i.e., in the second half of April into May, following the flights of *L.subfervens* and *L.pupula* at the type locality (Fig. 62).

##### Remarks.

A comparative assessment of *L.rubritegula* relative to *L.basistriga* is provided in Matson and Wagner (2017).

#### Lactura nalli

Taxon classificationAnimaliaLepidopteraLacturidae

Matson & Wagner sp. n.

http://zoobank.org/D25568AD-1682-4C9F-8198-A403AC074DFE

Figs 2 , 9–14 , 21 , 23 , 34 , 38 , 48 , 51 , 52 , 60 , 63 , Table 1

##### Diagnosis.

Adult *Lacturanalli* are similar to *L.basistriga*, and in some cases indistinguishable. The following generalizations can be made when comparing series of adults: when the red basal streak is present, it is almost always shorter, usually ending before the most basal antemedial spot; the antemedial wing spots tend to be more reduced; and the hindwings are more roseate and given to pink, while those of *L.basistriga* are more orange-red in hue. *L.nalli* can be distinguished from *L.rubritegula* by the absence of red tegular scales (that are visible in dorsal view). Like *L.basistriga*, many individuals can be differentiated from *L.rubritegula* by a reduced upper postmedial spot and the convex arc facing the termen of the three lower postmedial spots. *L.nalli* can be separated from *L.subfervens* by the absence of the scattered flecking of red or brown scales over the forewing. Female genitalia overlap with *L.basistriga* but differ from other *Lactura* in having 4–5 anterior whirls present in the spiraled ductus bursae. Larvae can be immediately distinguished from those of other North American *Lactura* by their green semitransparent dorsum lacking any black or white markings, which is in stark contrast to the dark dorsum of *L.basistriga*.

##### Description adult.

(Figs 2, 21) Forewing length: male: 8.5–9.5 mm (n = 21); female: 9–10 mm (n = 8). Body salmon red. **Head.** Shiny, white decumbent scales over vertex and frons. Labial palpi fuscous red, straight or slightly porrect. Antenna filiform, 0.6 length of forewing; shiny, white decumbent scales over scape and basal 1/3, transitioning to fuscous to orange scales; fuscous beneath. **Thorax**. Patagium mostly white; red basally, most apparent around perimeter of eye. Tegula white with small ventral red basal patch. Subtriangular medial mesothoracic red spot flanked posterolaterally by ellipsoid to bar-like red spots. Coxa and femur with reddish mesal surfaces, distal and ventral surfaces with admixture of white and red scaling; tibia given more to white scaling; protarsus mostly red, meso- and metatarsus given to more white scaling. **Forewing.** Pearly white, with seven blood- to mahogany-red spots in oblique antemedial and postmedial series; without scattered dark scales (of *L.subfervens*); antemedial row with three spots, postmedial row with four spots. Lower three spots of postmedial row forming straight line or more commonly a convex arc (closed to termen); on average, spots more reduced than those of *L.basistriga* (when comparing series). (These same three spots often form concave arc, open to termen, in *L.rubritegula* due to a more distal displacement of the lowermost spot.) Basal, subcostal red dash, typically ending before basal antemedial spot; this dash always present in males but usually absent in females. Basal red scaling along costa often narrows and ends before antemedial spots. Underside light red with pale fringe scales. **Hindwing.** Pinkish red in fresh individuals; dull red below but paler along inner margin. **Abdomen.** Dorsum and sides dull orange-red; venter rusty white. Two pairs of subventral intersegmental hairpencils (with 40–60 androconial scales) inserted between A6 and A7, and A7 and A8 (Fig. 34). **Male Genitalia** (Fig. 23) (n = 1). As in *L.basistriga*. **Female Genitalia** (Fig. 38) (n = 1). As in *L.basistriga*.

##### Description of living final instar.

(Figs 48, 51) Ground color glossy green with green semitransparent dorsum, lacking pale addorsal stripes and black dorsal pigmentation of *L.basistriga*, and brick-red to black dorsal coloration of *L.rubritegula*. Alimentary canal visible as a pseudo-middorsal stripe (viewed through transparent heart). Thick white subdorsal stripe punctuated by raised yellow warts with D2 setae borne from apex. Subdorsal warts on A9 yellow, never orange-yellow as in *L.basistriga* or black as in *L.rubritegula*. Two, wavy-edged, pale, supraspiracular stripes extending from T1–A8. Prothoracic shield well differentiated, medially divided, partially melanized, with little pigment deposition along its anterior and lateral margins. Head brown, partially retracted into prothorax.

##### Type material.

**Holotype male**, dry pinned, TX: Starr Co., Falcon Heights (26.5585N, 99.1220W), 05 March 2018, Berry Nall coll., Deposited at USNM, Washington D.C., USA. **Paratypes adults** (18♂, 8♀): TX: Starr Co., Falcon Heights (26.5585N, 99.1220W), 25 February 2018 – 19 March 2018, Berry Nall coll., BBN18#01, BBN18#05, BBN18#06 (6♂, 5♀) (UCMS); TX: Starr Co., Falcon Heights (26.5337N, 99.1059W), Berry Nall coll., BBN14#06c, (ex-ova; 30 March 2014) emerged 11 June 2014, reared on *Sideroxyloncelastrinum*, genitalia slide #TAM–2017–014, CO1 Barcode DLW–000486 (1♂) (UCMS); TX: Starr Co., Falcon Heights (26.5337N, 99.1059W), Berry Nall coll., BBN14#06a, (ex-ova; 30 March 2014) emerged 27 May 2014, reared on *Sideroxyloncelastrinum*, Voucher Code TAM0011 (1♀) (UCMS); TX: Starr Co., Falcon Heights (26.5337N, 99.1059W), 19 March 2014, Berry Nall coll., genitalia slide #TAM–2017–013, CO1 Barcode DLW–000569 (1♀) (UCMS); TX: Starr Co., Falcon Lk. (26.559N, 99.125W), 19 March 2018, Jim Vargo coll. (11♂,1♀) (USNM) (TAMUIC).

##### Other material examined.

**Adults.** TX: Starr Co., Falcon Heights (26.5585N, 99.1220W), 27 February 2018, Berry Nall coll. (1♂); TX: Starr Co., Falcon Heights (26.5585N, 99.1220W), 06 March 2018, Berry Nall coll., BBN18#07 (1♂) **Larvae.** TX: Starr Co., Falcon Heights (26.5337N, 99.1059W), ex-ova from female 25 February 2018 – 6 March 2018, BBN18#01, BBN18#05, BBN18#06, BBN18#07, Berry Nall coll., (n~60) (UCMS).

##### Etymology.

We name this new species after our colleague Berry Nall who provided the majority of type material, reared four clutches of larvae, and first photographed the larva.

##### Distribution and biology.

So far as known, *L.nalli* and *L.basistriga* are allopatric in south Texas, with *L.basistriga* being limited to Tamualipan communities of the lower eastern Rio Grande Valley, and *L.nalli* restricted to the Chihuahuan scrub areas of the western end of the Valley (Fig. 52). The east and west ends of the Rio Grande Valley differ substantially in habitat: the east end is subtropical with palm forests and dry to mesic scrub thickets, while the west end (e.g., Starr County) is drier and more typical of Chihuahuan desert communities. Many species of plants and animals occur at one end of the Valley and not the other. While our collections are limited for *L.nalli*, peak activity of *L.nalli* appears to be from February through March when spring rains are frequent and *Sideroxyloncelastrinum* is flushing new leaves (Fig. 60).

### Molecular Data

Our phylogenetic analyses of a multi-gene dataset based on seven genes (Fig. 63) suggest that the six species resident north of Mexico fall into two well supported groups with *L.atrolinea* and *L.pupula* forming a group sister to the remainder: *L.basistriga*, *L.nalli*, *L.rubritegula*, and *L.subfervens*. The latter clade, in turn, divides into two groups with *L.subfervens* sister to the three other Nearctic members of the genus (*L.basistriga*, *L.nalli*, and *L.rubritegula*). Relationships among these remain unresolved with (uncorrected) pairwise distances among the three cryptic taxa averaging ~4.5 percent. Average pairwise distance among all sister taxa in the phylogeny was ~5.9 percent. We caution that our phylogenetic analyses consider only US species, and that many relevant Mexican sister taxa (including apparent sister species, based on barcode data in BOLD) were not represented. For example, the omission of the apparent Mexican sister taxa of *L.pupula* and *L.atrolinea*, inflates the average distance between sister taxa that we report. While a few loci did not amplify (Table 1), phylogenetic resolution improved when loci without full coverage were included (Wiens and Morrill 2011).

## Discussion

*Lactura* has challenged some of North America’s top systematic microlepidopterists, including August Busck, Harrison Dyar, and Ronald Hodges. Our contributions to the biosystematics of this group were kick-started by larval material and CO1 data for initial sorting. Both data sources were quintessentially important for *L.nalli*, for which no definitive adult characters are yet known. The nuclear data presented here were the last data to be added, and thus acted as a corroboratory data source, affirming the taxonomic decisions that we made based on larvae, life history information, CO1 data, genitalic characters, and distributional data.

As the taxonomic understanding of this group is untangled, it is likely that North American members of *Lactura* will be relegated to a new or different genus. The type-species *Lacturadives* (Walker, 1854) is a striking orange and black Australian moth with greater than ten percent (uncorrected) barcode dissimilarity from North American members of the genus. Presently, more than 80 species of *Lactura* are distributed worldwide and include an array of seemingly unrelated phenotypes (Heppner 1995, Roskov et al. 2018). Global barcode data suggest that current *Lactura* classification is unnatural and in need of a modern revisionary treatment.

All known species of North American *Lactura* are specialists on the host-plant genus *Sideroxylon* (Sapotaceae). *L.pupula*, *L.rubritegula*, and *L.subfervens* occur in sympatry in central Texas where they use *Sideroxylonlanuginosum*. We have found caterpillars of *L.pupula* and *L.subfervens* feeding alongside one another on early spring foliage; *L.rubritegula* occurs later, flying only at the tail-end of the flights of its congeners (Matson and Wagner 2017). *L.pupula* and *L.subfervens* co-occur widely through the Gulf States, where they share the same host species. In southern Texas, *L.basistriga* and *L.atrolinea* share *Sideroxyloncelastrinum* in sympatry. Both moths appear to share the same niche and are active synchronically – a common phenomenon among lepidopterans (Wagner 2005, Wagner et al. 2002, 2011, Nakadai and Murakami 2015, Nakadai and Kawakita 2017, Matson unpubl. data) that appears to challenge the generality of Gause’s competitive exclusion principle as a fundamental paradigm in the organization of lepidopteran communities.

Phelan and Baker (1987) hypothesized that male scent-emitting structures in moths are more likely to evolve among closely related sympatric Lepidoptera species that share a common host (because of a higher probability of mating mistakes) than closely related taxa with different hosts. A phylogenetically rigorous example supporting their hypothesis, drawn from a revisionary study of *Atlides* (Lycaenidae) butterflies, was recently published by Martins et al. (2018). Our finding of abdominal male-scent emitting organs in sympatric host-sharing *Lactura* (Figs 31–36) is also consistent with their thesis. It would be telling if tropical burnet moths feeding on novel hostplants or taxonomically (or geographically) isolated lacturids occurring elsewhere were shown to lack abdominal hairpencils or other androconial structures.

CO1 data were valuable in unmasking misidentifications, disambiguating cryptic lineages, and helping guide our fieldwork and genitalic dissections. Given our new understanding of this genus, the records in Barcodes of Life Database (BOLD) were in dire need of curation due to the confusion of *L.subfervens* and its phenotypic overlap with *L.basistriga* and *L.rubritegula*; as well as its failure to treat *L.psammitis* and *L.rhodocentra* as synonyms. Forty-six percent of the 116 North American *Lactura* specimens in BOLD were misidentified prior to the submission of Matson and Wagner (2017).

Evolution advances at different rates across the various behavioral, ecological, molecular, and morphological axes used to adjudicate taxonomic decisions (e.g., adult color/pattern, male and female genitalia, larval morphology, DNA, courtship behaviors, etc.). This revision of North American *Lactura* is a case in point that speaks to the value of larvae, life history data, and molecular analyses over the set of salient adult characters commonly used by moth systematists. Given the overlapping and seemingly indistinguishable adult phenotypes and male genitalic structures in *Lactura*, our findings serve as an endorsement of the value that larvae and molecular data hold for some lepidopteran groups. We venture that larval phenotypic and life history data will prove equally valuable in advancing our systematic understanding of the rich lacturid faunas of Mexico and the Neotropics.

## Supplementary Material

XML Treatment for Lactura

XML Treatment for Lactura pupula

XML Treatment for Lactura subfervens

XML Treatment for Lactura subfervens sapeloensis

XML Treatment for Lactura atrolinea

XML Treatment for Lactura basistriga

XML Treatment for Lactura rubritegula

XML Treatment for Lactura nalli

## Supplementary materials

Supplementary material 1 Table of localities Data type: occurrence This dataset is made available under the Open Database License (http://opendatacommons.org/licenses/odbl/1.0/). The Open Database License (ODbL) is a license agreement intended to allow users to freely share, modify, and use this Dataset while maintaining this same freedom for others, provided that the original source and author(s) are credited.Tanner A. Matson, David L. Wagner, Scott E. Miller
